# On the specificity of the recognition of m6A-RNA by YTH reader domains

**DOI:** 10.1016/j.jbc.2024.107998

**Published:** 2024-11-17

**Authors:** Julian Widmer, Andreas Vitalis, Amedeo Caflisch

**Affiliations:** Department of Biochemistry, University of Zurich, Zurich, Switzerland

**Keywords:** m6A, RNA binding, molecular recognition, molecular dynamics, computer simulation, Markov state models, binding kinetics, electrostatic interactions, force field, transition path theory

## Abstract

Most processes of life are the result of polyvalent interactions between macromolecules, often of heterogeneous types and sizes. Frequently, the times associated with these interactions are prohibitively long for interrogation using atomistic simulations. Here, we study the recognition of *N*6-methylated adenine (*m*^6^A) in RNA by the reader domain YTHDC1, a prototypical, cognate pair that challenges simulations through its composition and required timescales. Simulations of RNA pentanucleotides in water reveal that the unbound state can impact (un)binding kinetics in a manner that is both model- and sequence-dependent. This is important because there are two contributions to the specificity of the recognition of the G*m*^6^AC motif: from the sequence adjacent to the central adenine and from its methylation. Next, we establish a reductionist model consisting of an RNA trinucleotide binding to the isolated reader domain in high salt. An adaptive sampling protocol allows us to quantitatively study the dissociation of this complex. Through joint analysis of a data set including both the cognate and control sequences (GAC, A*m*^6^AA, and AAA), we derive that both contributions to specificity, sequence, and methylation, are significant and in good agreement with experimental numbers. Analysis of the kinetics suggests that flexibility in both the RNA and the YTHDC1 recognition loop leads to many low-populated unbinding pathways. This multiple-pathway mechanism might be dominant for the binding of unstructured polymers, including RNA and peptides, to proteins when their association is driven by polyvalent, electrostatic interactions.

The discovery of ever more varied, polyvalent interactions between macromolecules has revealed living cells to be governed by highly connected, malleable interaction networks (1, 2). While some level of abstraction will always be desirable if not required for human comprehension, the combined phenomena of intrinsic disorder in biopolymers, compartmentalization, and weak, transient interactions fuel the belief that reductionist approaches fall short of capturing all but the most idealized processes with sufficient accuracy. Crucially, mechanisms relevant for the fine regulation *in vivo* arise from interactions both between different copies of macromolecules (*e.g.*, protein-protein-interactions, aggregating proteins) as well as different classes of macromolecules (*e.g.*, protein-RNA-complexes).

In an attempt to offer mechanistic explanations as well as realistic illustrations for such complex interactions, molecular dynamics (MD) simulations can be a valuable tool. It has been a recent focus of MD to model larger, more complex molecular (sub-)systems of heterogeneous composition (3, 4, 5). Modern iterations of force fields (FFs) have been tweaked to model intrinsically disordered proteins more accurately (6, 7), and simulations of very large systems like intracellular condensates (8) or virus capsids (9, 10) have been attempted. RNA-protein complexes have received some attention as a particularly challenging type of molecular system (11, 12, 13): the binding is often sequence-specific yet driven in part by electrostatic complementarity and involves single-stranded RNA that is largely unresolved experimentally. As the systems modeled grow in size and in compositional complexity, so does the range of relevant timescales. This is often addressed by specialized enhanced sampling techniques, which tend to pose additional challenges during analysis.

The reversible, posttranscriptional modification of mRNA is one among many examples of the expansion to the regulatory pathways available to the cell (14). These so-called epitranscriptomic modifications have received attention for their implication in various cellular processes (15). A chemically often rather subtle modification can specifically alter the interaction properties of RNA with proteins, which is a pervasive interaction in biology. Unsurprisingly, the dysregulation of epitranscriptomic modifications can be accompanied by disease (16), making modified mRNA a target for therapeutic endeavors (17).

The installation of a methyl group on *N*6 of adenine (*N*6-methylated adenine, *m*^6^A) is the most common RNA modification, and it is embedded in the consensus DRACH-motif (D = G/A/U, R = G/A, H = A/U/C) which is recognized by the “reader”-protein YTHDC1 (18). The GGACU consensus sequence originates from the writer-complex responsible for the installation of the *m*^6^A modification. However, biochemical measurements have determined a certain degree of sequence-specificity with regards to the reader proteins. Specifically for YTHDC1, from oligonucleotide models, the mutation of both flanking positions to A or the deletion of these positions from pentanucleotides leads to a loss in affinity of 1 to 2 orders of magnitude (19, 20). This is comparable to the factor of roughly 50 by which affinity drops upon demethylation, as observed in a slightly different RNA sequence context (21).

The choice of FF for the simulation of biological systems is pivotal: each FF corresponds to a different set of parameters for partially empirical interaction functions, *i.e.*, approximations. These approximations might be more or less adequate for different combinations of FFs and systems. For an RNA-protein complex, the descriptions of both protein and RNA can lead to systematic FF errors, which have been characterized separately. It is fair to say that RNA FFs have received less attention and proven more challenging than protein FFs as highlighted by recent evaluations (22, 23), despite claims to the contrary (24). Divalent ions and descriptions of base stacking are particular areas of concern. As a consequence, various RNA parameter revisions have been proposed to alleviate specific FF deficiencies (25, 26, 27, 28). Alternatively, researchers have developed reweighting schemes such that MD-derived populations be made more consistent with NMR measurements *a posteriori* (29, 30). Compounding the FF issue, protein-RNA interactions are inherently hard to explore (31, 32), and FF evaluations usually have to focus just on maintaining experimentally observed, bound structures (33).

The interplay of parameters can both exacerbate and compensate systematic errors, but this is not easily known or tested. As a result, protein parameters that are appropriate for a protein in water may result in imbalanced interactions in the presence of nucleic acids. The water model itself is also known to affect the solvated macromolecules even within a FF family (6, 24, 34, 35). Systematic evaluations of FF performance will generally be out of reach for the (at least) ternary system of solvent, protein, and RNA. Thus, we argue that simulations will have to rely on proper controls instead, such as comparing methylated to nonmethylated RNA with the same FF or including variations on the RNA’s consensus sequence. The Amber χOL3 FF is sometimes cited as the most appropriate for atomistic simulations of RNA (36), although systematic comparisons are not common (22). For simulations of RNA-protein complexes, the consensus is even less clear: for example, comparing Amber and CHARMM variants has led to some contradictions in describing the binding of proteins to mRNA containing *N*6-methyladenine (19, 37).

In this work, we aim to contribute to the effort of extending the scope of molecular systems studied by MD by investigating one of the epitranscriptomic modules. We first investigate the behavior of the oligo-RNAs GGACU and GG*m*^6^ACU in water, which has the potential to reveal the nature of the unbound state, along with possible sources of FF-based disagreements. Further, we use an adaptive sampling scheme to scrutinize the sequence dependence of *m*^6^A recognition by a YTH domain, which, for the purpose of this manuscript, can be regarded as a prototypical reader module (Fig. 1). We demonstrate that modern high-performance computing architectures in conjunction with optimized MD engine code and adaptive sampling methods permit interrogation of RNA unbinding from the YTH-domain. We further gauge the relative importance of the cognate sequence compared to the methylation state of the RNA. Lastly, we ask whether dominant unbinding pathways can be identified that permit the assignment of rate-limiting steps to a given sequence and methylation state. We note that a model-derived ground truth is not available, which prevents an assessment of our sampling strategy with respect to systematic errors. To circumvent this, experimental knowledge is often brought in for comparison, and we do the same here, but this has a number of caveats (38, 39). Thus, in first instance, the analysis of such complex data sets calls for unsupervised, data-driven techniques.

**Figure 1:**
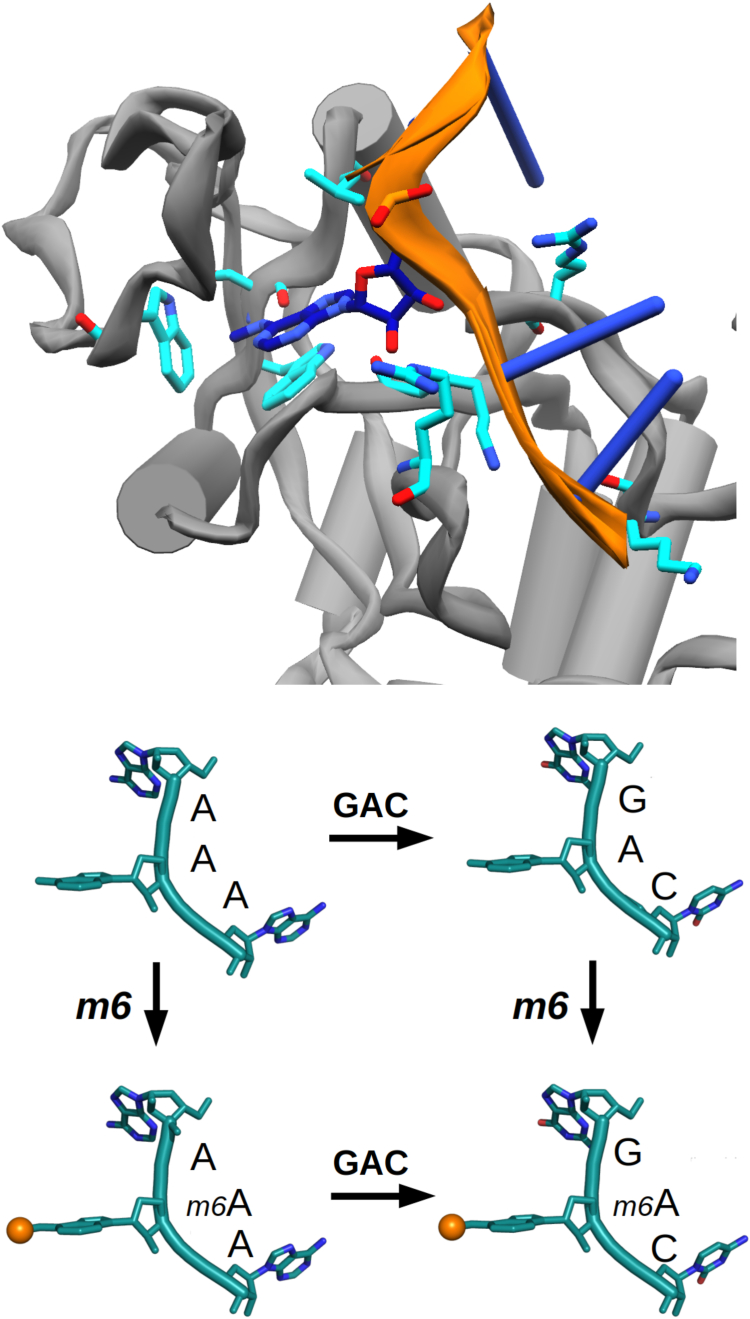
**Cartoon visualizations of the studied system.***Top*: PDB entry 4R3I forms the basis for the unbinding studies and FF comparisons presented here. The aromatic cage, including Ser378, and basic protein residues that bind the RNA in the crystal structure are highlighted in *cyan*. The RNA itself is shown in *orange* and *dark blue*. *Bottom*: The four trinucleotides subject to unbinding simulations from the YTH protein domain. G*m*^6^AC represents the canonically bound target, AAA is the designated negative control. The methyl-group of *m*^6^A is represented as an *orange sphere*. FF, force field; *m*^6^A, *N*6-methylated adenine; PDB, Protein Data Bank.

## Results

### Conformational ensembles of GGACU and GG*m*^6^ACU in water

We first aimed to determine the impact of methylation on structural features of monomeric RNA in MD simulations. Furthermore, we wanted to gauge whether two FFs, Amber and CHARMM, differ in these properties. The conformation of the pentanucleotide GGACU is essentially described by 31 dihedral angles. The first two principal components (PCs) constructed from those dihedral angles (40) along the simulation trajectories capture most of the dynamics of the phosphoribose backbone (Fig. S2). Restricting ourselves to the monomeric state and short chains with the consensus sequence makes the simulations tractable but eliminates a number of avenues in which methylation can act in reality (41, 42, 43), which is a caveat.

**Figure 2:**
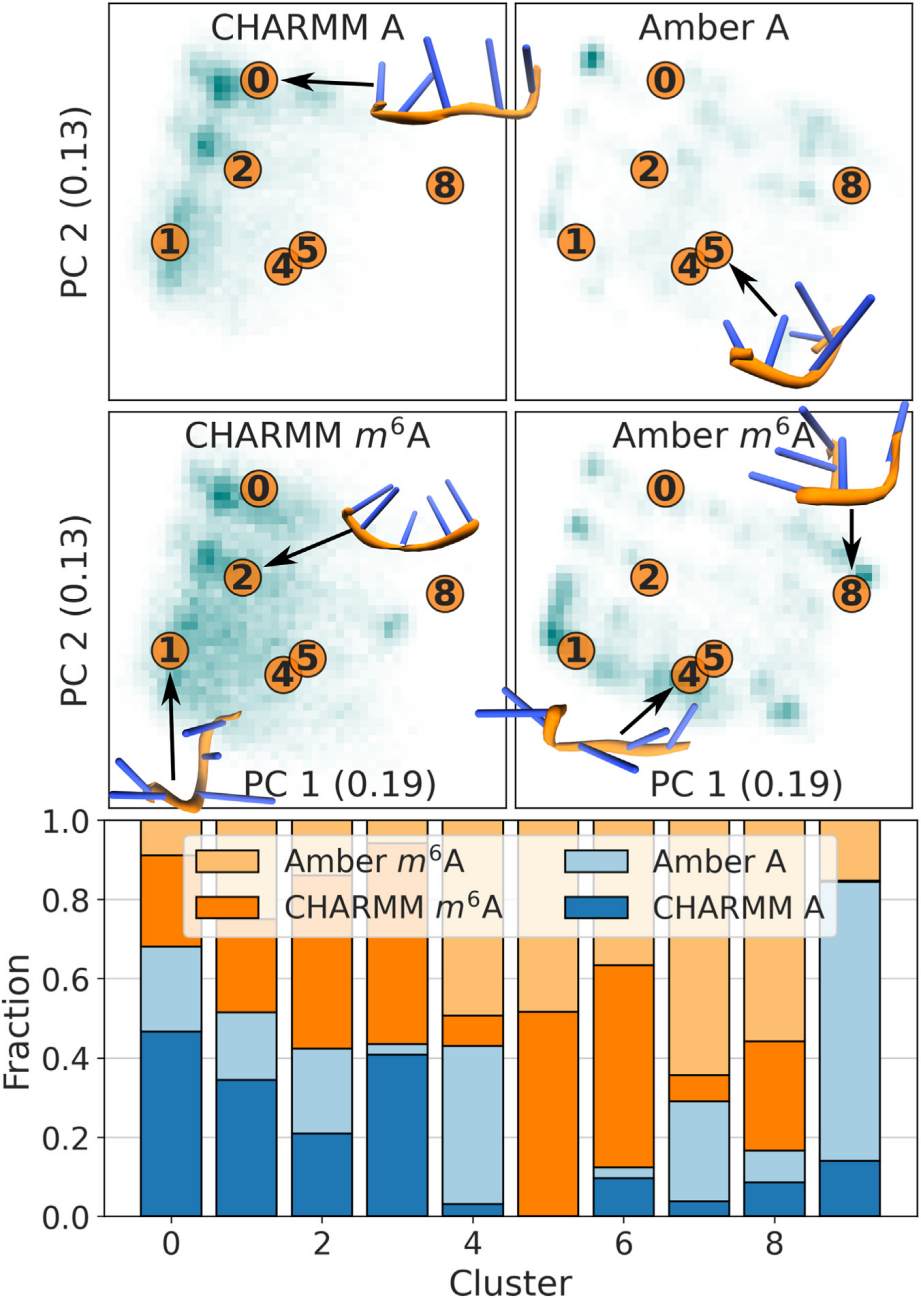
**Structural properties of GGACU in water in methylated and unmethylated states.***Top*: 2D-histogram of the projection onto the two first PCs. Some of the most populated clusters are marked on the projection. The components’ fraction of the total variance is noted in parentheses. *Bottom*: relative population of the 10 most populated clusters across the four setups. PC, principal component.

Amber and CHARMM lead to populations of roughly the same area in PC space (Fig. 2, top panel). However, simulations under Amber lead to smaller, more sharply delineated minima compared to CHARMM. This is true not only for the shown components, but coupling between dihedral angles as measured by mutual information was overall markedly tighter in Amber (Fig. S3). In addition to the more structured free energy landscape in Amber, methylation has a distinct effect on both FFs. In CHARMM, *m*^6^A further broadens the existing minima with the main modes remaining heavily populated (Kullback-Leibler divergence: 4.7). Amber, in contrast, shifts the majority of the main configurations to minor modes upon methylation, which is a more pronounced shift (Kullback-Leibler divergence: 15).

To make the notion of major shared states between FFs explicit, a tree-based clustering is applied to the top 10 PCs (83% of variance; Fig. S2) for a systematic assignment of states. The clustering is agnostic to the methylation state and is applied to all samples jointly, thus identifying each FF’s preferential states. Moreover, the clustering reports on how methylation changes the conformational preference for a given FF. Figure 2 (bottom panel) indicates that the 10 most populated states are almost all shared, both between FFs and between methylation states, albeit often with a strong imbalance. The four most populated clusters, which accumulate roughly one half of the total weight (Fig. S4), are dominated by configurations resulting from CHARMM36. Clusters 4 to 8 are populated preferentially by methylated RNA from both FFs. Beyond the four largest states, it is expected that the less populated clusters consist predominantly of Amber-specific conformations, as seen for clusters 4 or 7 to 9. Each of them accounts for <5% of sampling, which corroborates the notion that Amber introduces multiple, sharply separated minima. Part of this behavior may be explained by Amber favoring base stacking (Fig. S5), which is an important feature of nucleobases, but is modeled empirically, mostly through Lennard-Jones interactions, in classical FFs (44).

**Figure 3:**
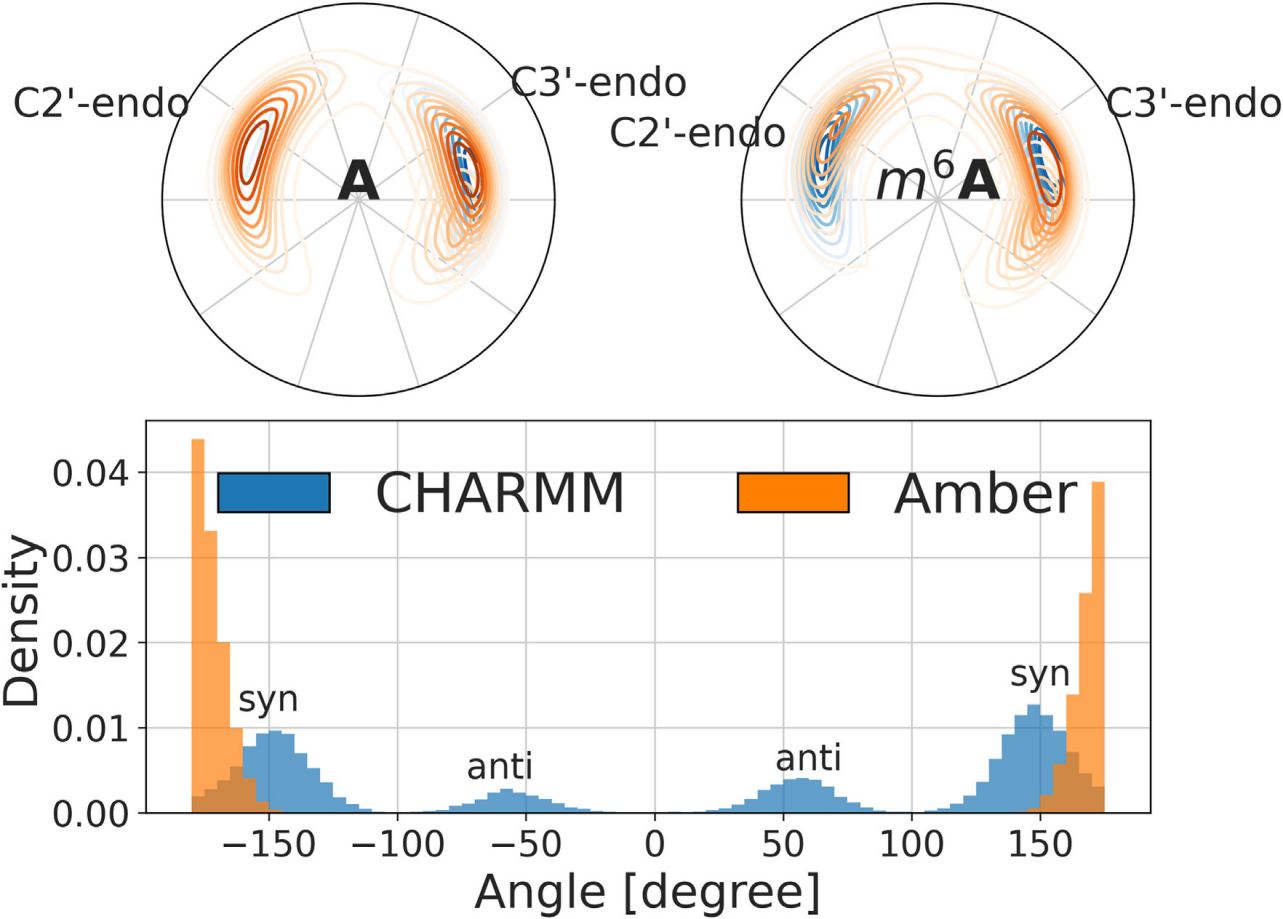
**Configurational details of the central adenine in the absence of protein.***Top*: distribution of sugar pucker of the central adenine in GGACU or GG*m*^6^ACU in the absence of protein. *Bottom*: distribution of the dihedral angle of *m*^6^ relative to the purine ring (CM6-N6-C6-C5) of the central adenine in GG*m*^6^ACU in the absence of protein. *m*^6^A, *N*6-methylated adenine.

The differing effect of the methylation also manifests itself in the sugar pucker of adenosine, which, from NMR data, is normally in a ns-regime for interconversion (45, 46). Simulations are generally able to explore this equilibrium, at least locally and for unmodified RNA (47). However, significantly slower timescales are known (48), and the pucker transition may thus, directly or by its coupling to nearby dihedral angles, contribute to long timescales of conformational evolution in RNA-protein simulations. In Amber, both major modes, C3′-endo and C2′-endo are populated, as is canonical for RNA. Methylation shifts the equal populations such that the C3′-endo configuration is preferred slightly. CHARMM, on the other hand, permits C2′-endo configurations only for *N*6-methylated adenine (Fig. 3, top panel). Justifying this coupling is difficult since the methylation occurs on a distant site compared to the ribose backbone. While the population of the major sugar pucker modes of *m*^6^A is similar under both FFs, the differences matter since we use unmethylated RNA as a reference in simulations (Experimental procedures: PIGS simulations of unbinding). The treatment of the dihedral angle of *m*^6^ itself differs as well: on the timescales probed here, no transitions from the initial, and preferred (49, 50), *syn*-configuration occurs in Amber. CHARMM permits such transitions more readily, even overestimating the *anti-*population compared to experimental values (42) (Fig. 3, bottom panel), while also recognizing the partial pyramidal structure at *N*6, which seems to be overlooked by Amber.

In choosing a nucleic acid FF, ambivalent trends are a common problem (36). Here, we would prefer AMBER for a lesser impact of *m*^6^A overall but CHARMM for lower free energy barriers and better *N6* geometry. This is confounded by the fact that *m*^6^A is a FF addition, which means that the parameters (37, 51, 52) are less tested and often fail to be updated as the parent FF evolves, leading to eventual deprecation. Based on Figures 2 and 3, it is also unclear how much *m*^6^A parameters are actually linked to the parent FF (Supplementary Methods, Parameterization of m6A). For example, the largest clusters contain more examples that are predominantly methylation-specific than that are FF-specific. This weak “heritage” is also directly visible in the partial charge parameters.

In summary, global and local structural descriptors and measures of similarity are leveraged for a detailed view of differences in the conformational ensembles of GGACU and GG*m*^6^ACU in two FFs. Analysis based on dihedral angles reveals that major clusters are shared across all four combinations. Globally, Amber leads to a more structured free energy landscape and tighter coupling between nearby angles than CHARMM. Beyond the backbone, *m*^6^A can transition between *syn-* and *anti-*configurations, but only in CHARMM. At the same time, Amber is more permissive and consistent with respect to transitions between configurations of the sugar pucker. We conclude that these details matter and must be kept in mind throughout: the methylation of RNA is certain to have some impact on the structural properties of single-stranded, unfolded, monomeric RNA, but there is no consensus on what exactly these changes should be. This is in addition to the neglection of the more complex unbound-state effects alluded to above. Structurally, there is evidence from various *in vitro* data that *m*^6^A must be in a loop-like or single-stranded region for binding to occur (53).

### Adaptive sampling efficiently achieves trinucleotide unbinding

**Figure 4:**
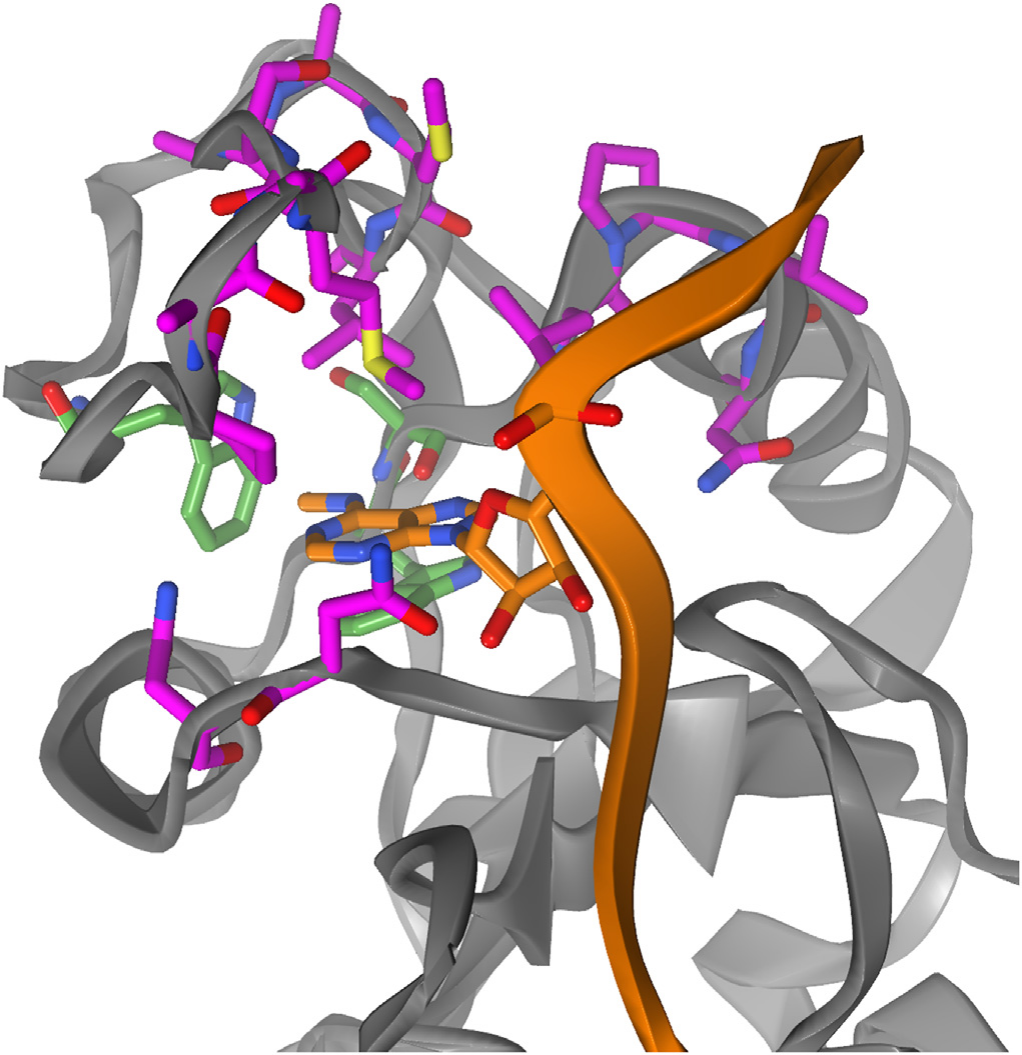
**Set of residues deemed important for maintaining the bound pose.** YTHDC1 as seen in 4R3I accommodates methylated adenine in its aromatic cage (*green sticks*). Those protein residues that are highlighted as *magenta sticks* are, along with the RNA (in *orange*), subject to diversification in PIGS. This includes Leu380, which replaces the third residue of the aromatic cage. Specifically, the *ϕ*- and *ψ*-angles of the chosen protein residues and nonredundant phosphoribose backbone and glycosidic *χ*-angles contribute to the definition of the high-dimensional state space PIGS operates in. PIGS, Progress Index-Guided Sampling.

The YTH-domain is characterized by an aromatic cage, of which two tryptophan residues, Trp428 and Trp377, appear fully conserved. The third Trp residue is replaced by Leu380 in the DC1 protein (15). In the reference crystal structure 4R3I (20), the methylated *N*6 of the inserted adenine residue is in *syn*-configuration (Fig. 1). Inside the cage, the hydrogen on *N*6 forms a hydrogen bond with the backbone oxygen of Ser378. The adenine base is contained by a loop (residues Val429 – Leu439), closing the binding site from above. In particular, Met434 appears to clamp down on the inserted adenine. Residues Met438 and Lys437 may assume similar functions upon fluctuations of the loop (Fig. 4). Interestingly, both Ser378 and Met438 can be mutated to alanine while reducing affinity only by a factor of less than 3 (54). This suggests a certain malleability of parts of the binding site, which is consistent with the presence of multiple Gly, Ala, and a Ser-residue in the loop, conferring flexibility. The side chains of Arg404, Lys408, Lys361, Lys472, and Arg475 (highlighted in cyan on Fig. 1) form a basic patch for the accommodation of the RNA’s negatively charged phosphate backbone. Consequently, the bases flanking *m*^6^A are solvent exposed. In particular, residues C and U are reported to deviate only marginally from the crystal-like configuration in MD simulations on the scale of *μ*s. The guanine residues, on the other hand, do not form such stable ionic interactions and tend to be much more flexible (54).

The unbinding of GG*m*^6^ACU from YTHDC1 occurs on a timescale that currently seems intractable for conventional sampling. In Refs. (37, 54) as well as over the course of various adaptive sampling attempts in our hands, GGACU with and even in the absence of methylation remained fully bound in a largely stable pose. This behavior was observed for both Amber and CHARMM parameter families. We therefore restricted the scope of our investigation to trinucleotides instead of pentanucleotides. We anticipate that this reduction of complexity has a large benefit regarding the accessibility of timescales while simultaneously preserving the salient features of the system. In particular, we do not expect the bound pose of the modified base to be affected, which would be consistent with the high mutual similarity across many different RNA species bound to YTHDC1 (Fig. S1). The trinucleotides have two negatively charged phosphate groups in the backbone and thus reduced electrostatic interactions with the basic side chains with respect to the four negative charges of the pentanucleotides. At the same time, bases D and H are pruned from DR*m*^6^ACH, *i.e.*, bases which are known to exhibit low sequence specificity in experiments. We acknowledge that boundary artifacts may be introduced at the RNA’s 3′ and 5′ ends by this pruning. Even with this simplification of the system in place, the Amber FF did not yield unbinding of G*m*^6^AC from YTHDC1. In the following, we therefore focus on sampling obtained using the CHARMM36m FF. Three negative controls, AAA, A*m*^6^AA, and GAC, of which AAA is expected to be the weakest binder (Fig. 1), were chosen for investigating the relative impact of altering the consensus sequence or the methylation state on unbinding and are used to gauge the sampling efficacy.

The slow timescale of unbinding, even for trinucleotides, calls for enhanced sampling techniques. Progress Index-Guided Sampling (PIGS) is an adaptive sampling strategy free of Hamiltonian biases that exploits parallelism (55). It periodically terminates redundant replicas, meaning replicas that appear to be currently in the same area of phase space, and replaces them with rare configurations from the ensemble. PIGS thus promotes exploration of newly discovered configurations. The feature space for defining redundant configurations is a “hyperparameter” that can be chosen freely. As a general guideline, in the absence of known reaction coordinates, choosing a rich feature space has proved productive in our hands. Here, we settled on backbone dihedral angles (*ϕ* and *ψ*) of protein residues in the generous vicinity of the binding site along with most available RNA dihedral angles to define the feature space (Experimental procedures: PIGS simulations of unbinding). The flow of the algorithm, in particular how reseeding decisions are made, is explained in detail in “Supplementary Methods, Details on progress index-guided sampling (PIGS)”.

**Figure 5:**
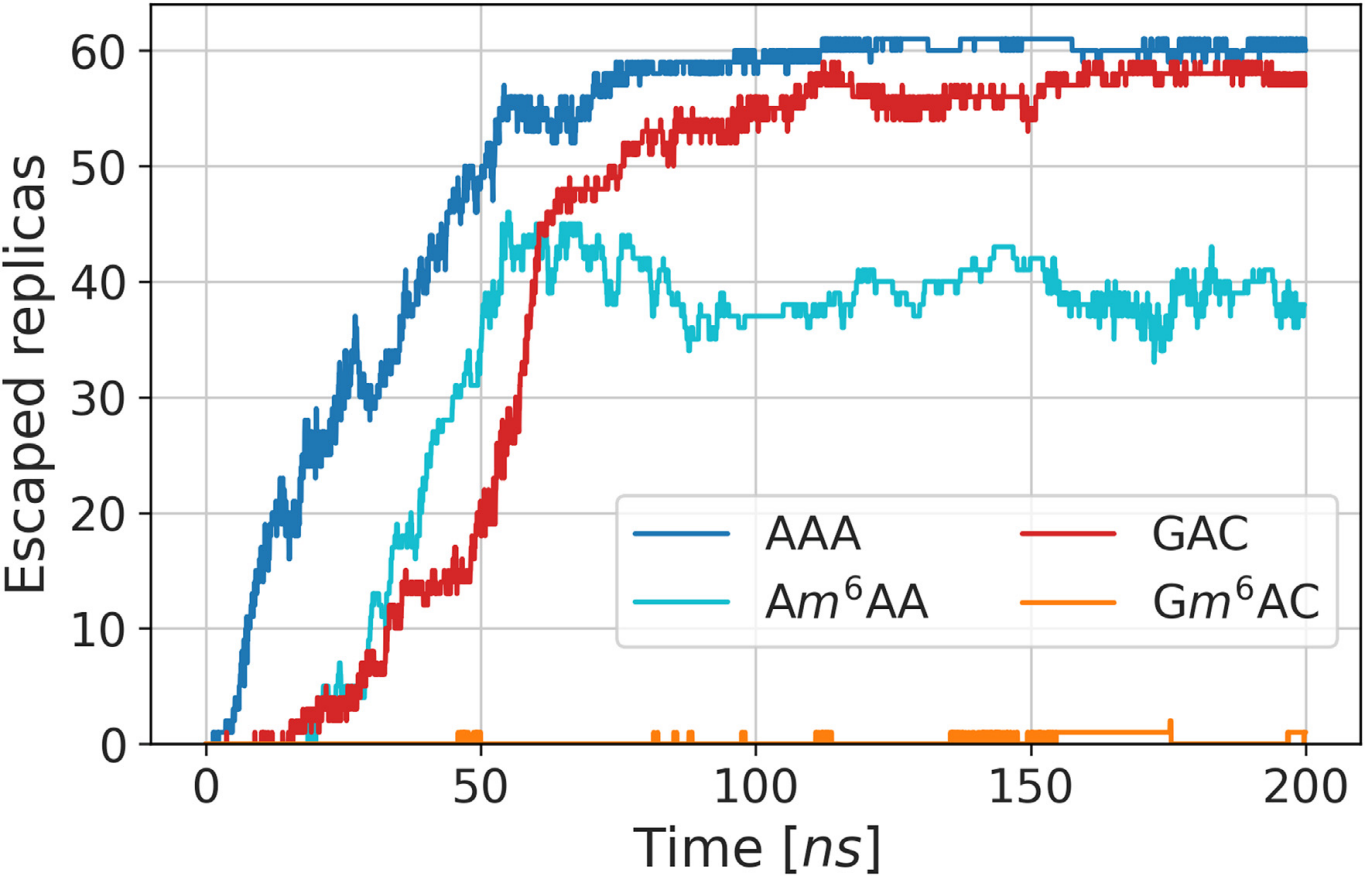
**Unbinding achieved by PIGS for each trinucleotide system.** For each point in the simulation time per replica, the number of replicas exceeding the 10 Å threshold between Trp428 and *N*6 is recorded. PIGS, Progress Index-Guided Sampling.

The residues chosen to comprise the feature space are highlighted in Fig. 4, and the majority of them make up the loop enveloping the aromatic cage. Monitoring the number of replicas exceeding a distance of 10 Å between *N*6 and Trp428, which is at the bottom of the cage, gives a coarse estimate of the extent of unbinding achieved over the course of the simulation (Fig. 5). This distance reports on whether the central adenine has left the binding pocket, which was exceedingly rare in previous attempts.

Indeed, PIGS succeeds in producing such unbinding in most replicas for the unmethylated RNAs, GAC, and AAA, within 100 ns. Consistent with its intended role as the weakest binder, AAA unbinds most readily from the YTH domain. One third of the replicas exceed the threshold after a few nanoseconds of PIGS. In contrast, conventional, brute-force sampling achieves the same extent of dissociation after 200 ns, suggesting that PIGS confers a speed-up of roughly one order of magnitude here. PIGS can be viewed as an entropically driven sampler that finds rare states through spontaneous fluctuations and a data-driven approach. As such, the acceleration it confers will be system-dependent and controlled by the specific balance of enthalpic and entropic contributions toward the process of interest.

Compared to AAA, the unbinding of GAC and also A*m*^6^AA is markedly delayed. It is a testament to both the complexity of the system and the quality of the models that the methylation appears to have a similar effect as mutating the sequence to the consensus sequence GAC of the epitranscriptomic writer complex. The bases of the adjacent nucleotides are largely solvent-exposed, which holds for a large number of experimentally determined, RNA-bound structures (Fig. S1). Thus, there is no intuitive mechanism to explain the pronounced specificity they confer. It is precisely the strength of MD that such nonobvious behavior can emerge, and it might be rooted in intermediate or unbound states. The simplicity of the distance-based reaction coordinate in Figure 5 of course obscures much of the information on the system-specific unbinding, and we present an analysis of unbinding process based on a more comprehensive description of the systems below.

As expected, when both consensus sequence and the methylation of *N*6 are introduced simultaneously, the complex is substantially more stable. While the threshold set here is exceeded a few times, this corresponds to only a single full unbinding event. In addition, several partial unbinding events are observed, where *m*^6^A retracts from the binding pocket, but the RNA’s backbone remains bound in the close vicinity of the binding site. We observed that departure of *m*^6^A from the binding pocket was accompanied by the outward rotation of the sidechains of two methionine residues, Met434 and Met438, and of Leu439. Indeed, the loop’s configuration is diversified substantially in a follow-up run designed to address the low event count for G*m*^6^AC, and this is described next.

We addressed the dearth of observed unbinding events in G*m*^6^AC by first constructing a preliminary Markov state model (MSM) for all four systems jointly. PIGS trajectories were subjected to a joint, sequence-agnostic clustering. An MSM was constructed with featurization and construction following the procedure detailed below and in “Experimental procedures: MSM construction and rate constant calculation.” One of the key quantities MSMs can predict is the committor probability *q*_*MSM*_: it is fixed for boundary states (0 and 1) and describes the chance, resolved per cluster, that a trajectory passing through a given cluster will reach the target state (here, chosen as unbound) before it reaches the source state (here, bound). Further details on the committor probability and how it relates to two-state modeling are found in “Supplementary Methods: Transition path theory (TPT).” Here, we selected clusters with $q_{MSM}<0.5$ in the joint model, meaning those that are more likely to reach the unbound state ($q_{MSM}=0$) before they reach the bound state ($q_{MSM}=1$), to constitute the starting snapshots for a follow-up run also relying on PIGS (Figs. S7 and S8).

This strategy exploits that PIGS achieves diversification of its selected feature space from the crystal structure in all systems (Fig. S6), but unbinding of RNA occurred only to a varying degree. The joint representation underlying the MSM permits exploration of unbinding pathways that were not (yet) observed to be productive in G*m*^6^AC but evidently succeeded in another system. This approach is conceptually supported by the close molecular similarity between the trinucleotides. That said, the joint committor value for the four different systems has no direct physical interpretation as an unbinding probability because it effectively allows the ligand to change identity along an unbinding pathway. Instead, our approach is a pseudo-Bayesian intervention to focus exploration on a subset of the diversified PIGS ensemble. The Bayesian aspect refers to the (biological) negative controls for inclusion of prior information in the sampling procedure: it provides guesses on what configurations may be intermediate to unbinding of G*m*^6^AC and explores them further using adaptive sampling.

Selecting 64 different starting configurations of G*m*^6^AC from 10 clusters to be evolved with PIGS ensures a structurally varied ensemble of snapshots (cluster centroids shown in Fig. S7, bottom panel). PIGS implies that not all of the starting structures can or should survive the simulation time of 100 ns (Fig. S8, top panel). Even so, this strategy is highly productive; the additional sampling (of smaller net size) resulted in meeting the specified distance threshold in >40 replicas (Fig. S8, bottom panel) with a wide range of (partially) unbound states visited.

**Figure 6:**
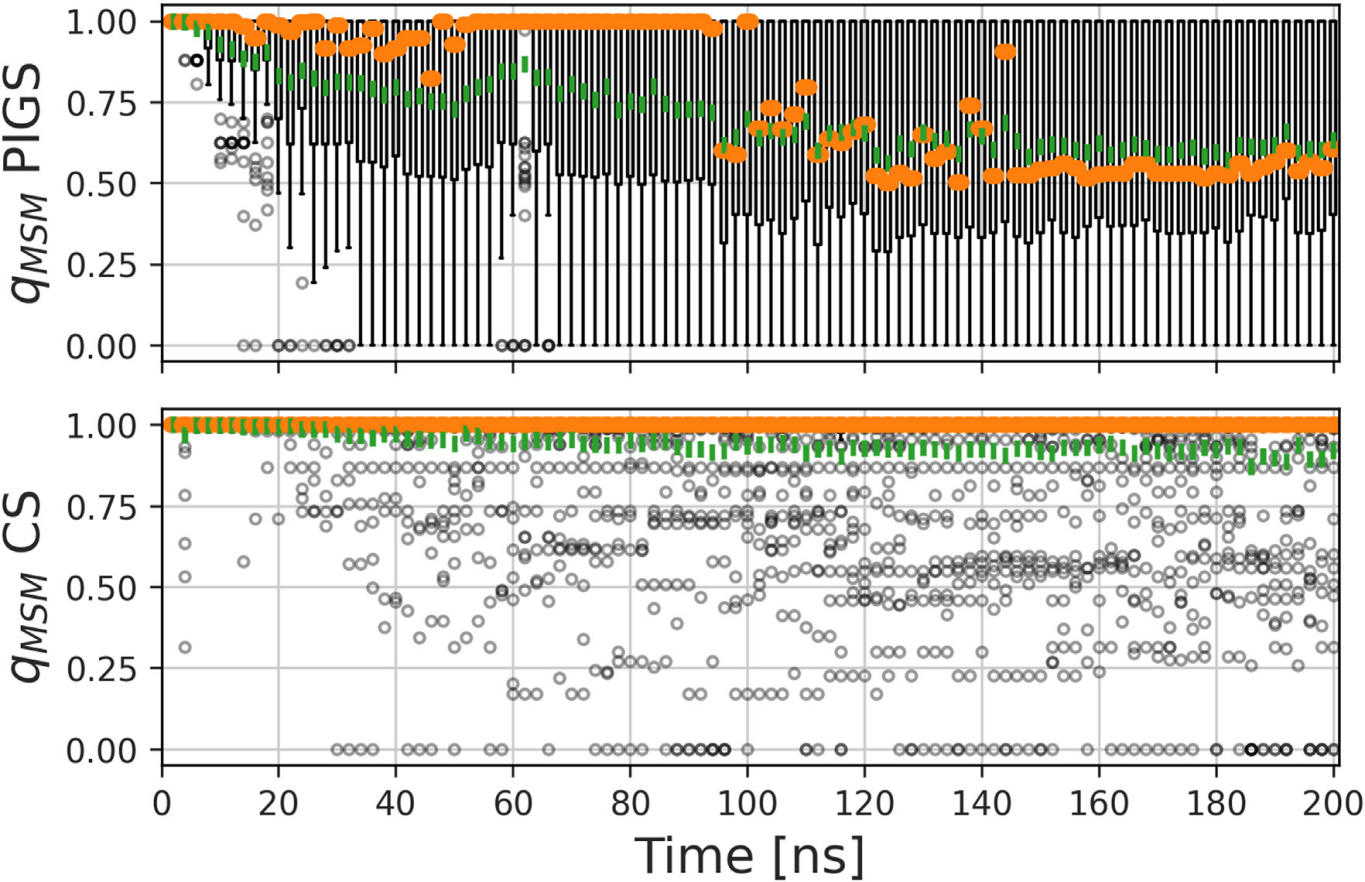
**The committor, *q***_***MSM***_**, calculated from a joint MSM for CS and PIGS specifically for the AAA system.** MSM parameters and boundary state definitions are provided as Supplementary Information. At each time point, a Tukey-style boxplot describes the distribution over the values of *q*_*MSM*_ across the 64 replicas. The median is marked in *orange*, the mean is shown in *green*. A replica-wise colormap of the same data is shown in Fig. S9. MSM, Markov state model; PIGS, Progress Index-Guided Sampling.

In addition to guiding the choice of new starting configurations, the committor can be exploited to assess systematically how efficient PIGS is at accelerating the unbinding, here using AAA as an example. PIGS itself is agnostic of explicit reaction coordinates, pathways, or boundary states; it relies solely on the feature space and a distance to measure the redundancy of replicas. The chosen dihedral angles are therefore not necessarily related to the committor, which is defined *a posteriori*. Indeed, this may be the reason why PIGS leads to varying degrees of unbinding for the four nucleotides in the first place. An appropriate reaction coordinate is not easily known *a priori*, and it is a strength of PIGS to not presume such knowledge. The distribution of committor values across simulation time between CS and PIGS reports that diversification of the chosen feature set does lead to a marked acceleration of unbinding (Fig. 6). On average, replicas remain close to the initial structure in CS throughout, while the whole range of committor values is sampled in PIGS from a simulation time of 40 ns onward.

**Figure 7:**
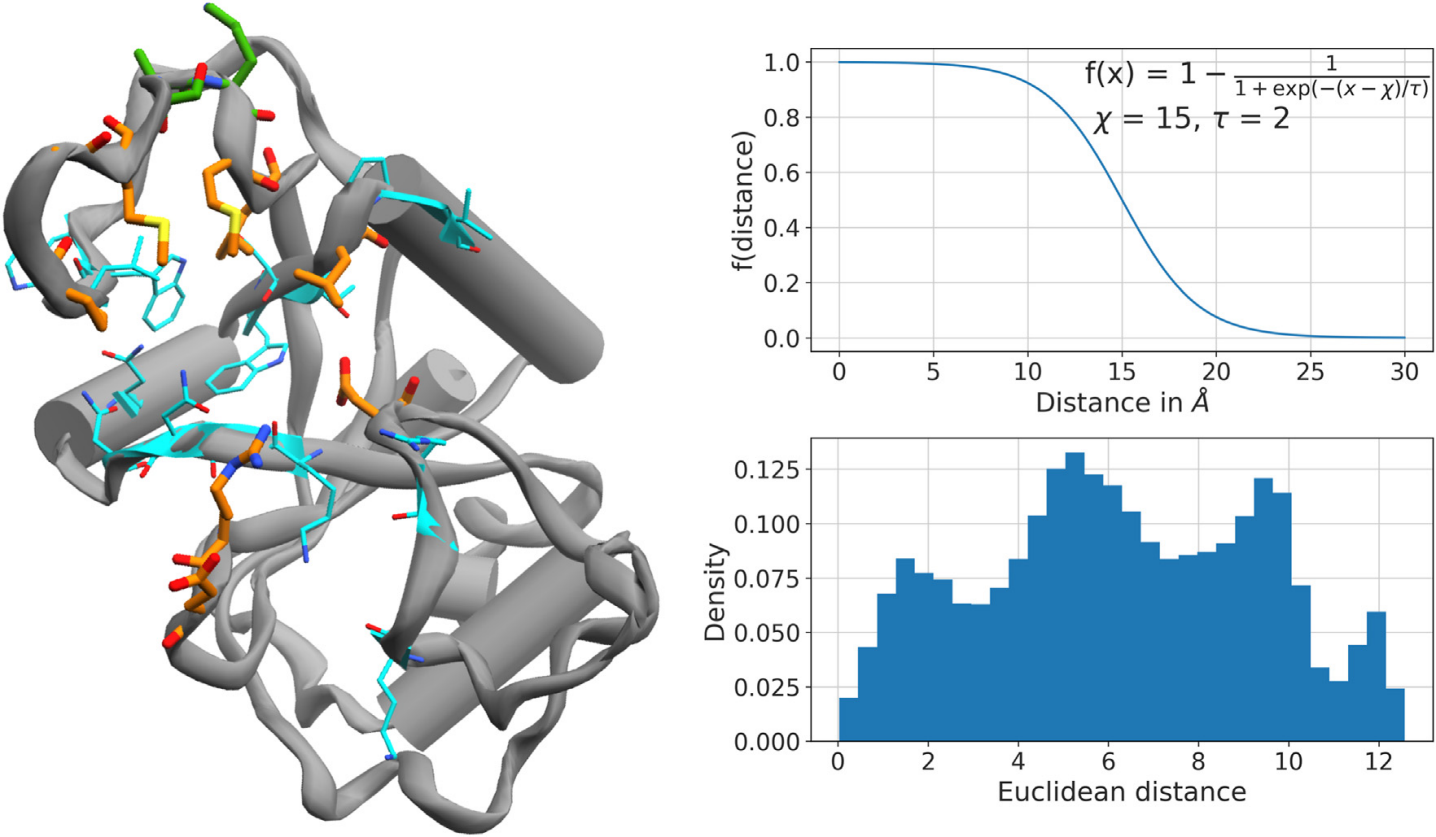
**Featurization of protein-RNA complexes.** Pairs of residues are chosen based on a contact map (Fig. S10). For protein residues, both the Cα and the most distant side chain heavy atom are selected. For RNA, the nitrogen forming the glycosidic bond, C4′, and O3′ are selected. From this, we form all pairwise combinations for every pair of residues meeting the cutoff. *Left*: protein residues involved in protein-protein distances are marked in *orange*. *Cyan* residues are involved in protein-RNA distances. *Green* residues feature in both intermolecular and intramolecular residue pairs. Naturally, all three RNA residues are included but omitted here for clarity. *Upper right*: the sigmoid function to transform interatomic distances. The parameter *χ* represents the midpoint of the sigmoidal curve and has units of Å, whereas *τ* is a smoothness parameter. *Lower right*: distribution of Euclidean distances for 10^5^ randomly selected pairs of configurations, which are featurized by the sigmoid-transformed interatomic distances. This highlights that the high-dimensional distance feature space is constructed in a way that offers enough contrast, *i.e.*, is able to resolve different states.

We next turn to a more fine-grained representation of the four PIGS trajectory ensembles. As is common for similar applications (56, 57, 58), we choose a rich set of distances based on a contact map of GAC for the first and last 25 ns of simulation time (Fig. S10). The contact map captures which residue pairs change their contact frequency most strongly upon unbinding of the RNA. The identified residue pairs encode the proximity of RNA and protein as well as the configuration of the protein itself (Fig. 7). The set of protein residues was pruned manually to exclude features likely to be inconsequential for unbinding, such as the N- and C-terminal helices, which undergo a slight change of orientation with respect to one another. The residues implicated in the remaining pairs intersect to a large degree with the set of residues subject to diversification in PIGS (*cf.* Figs. 4 and 7). The unbinding process can be expressed by casting the retained residue pairs as pairwise, interatomic distances. We apply a sigmoidal transform to force these distance-based features to focus on an intermediate distance regime (Fig. 7, bottom). This is done primarily to homogenize the unbound state. Discarding all but seven PCs (preserving 34% of variance) ensures that the relevant structural information is captured concisely.

**Figure 8:**
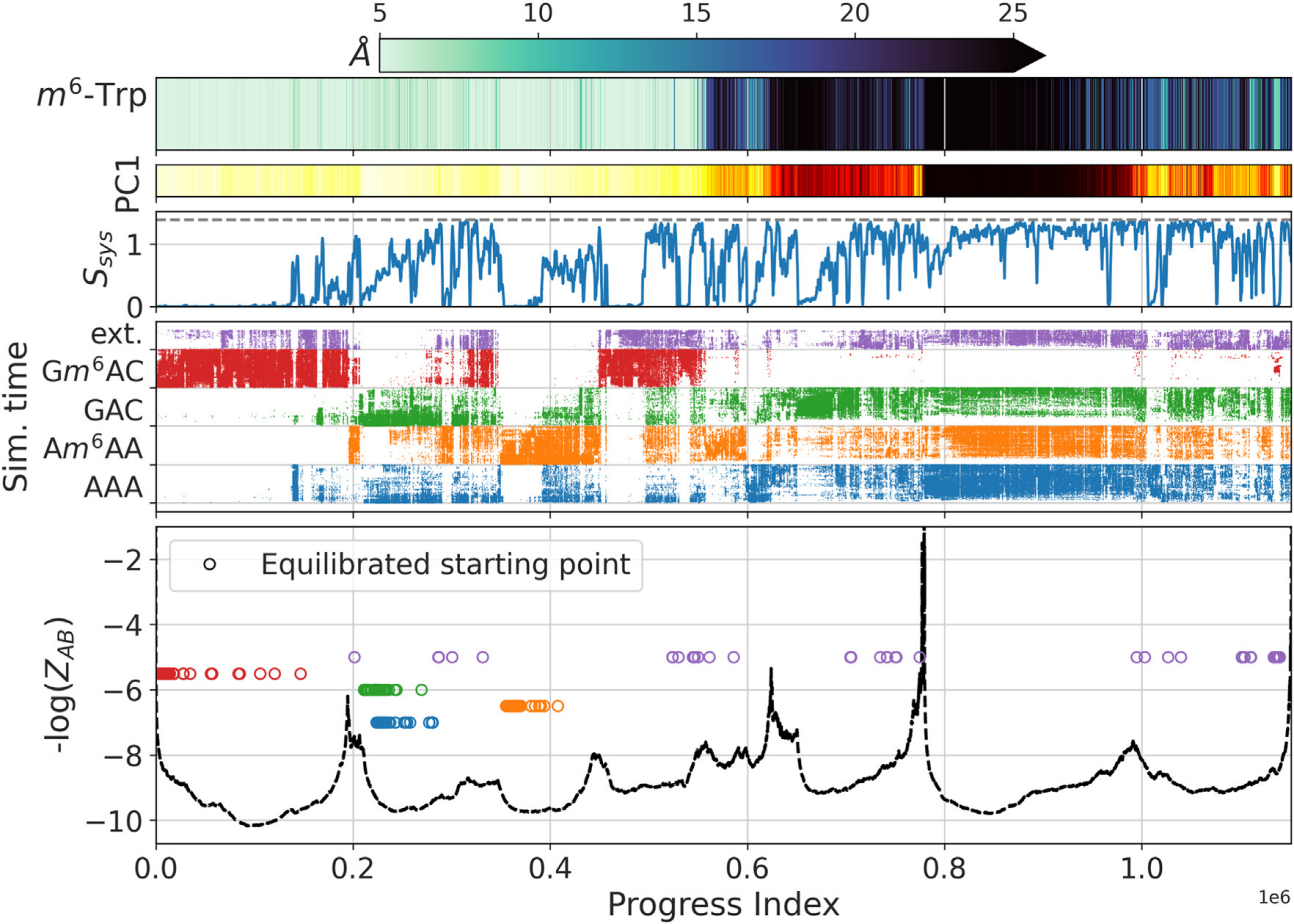
**SAPPHIRE plot of PIGS simulations for nucleotide unbinding from YTHDC1.***Top*: color-coded distance between *Cδ*_2_ of Trp428, which is part of the aromatic cage, and *N*6. *Second row*: the value of the first PC, which accounts for 16% of the variance, plotted as a color bar. *Third row*: entropy of the distribution of which system a snapshot originates from calculated in a rolling window of 1000 frames. The upper bound for perfect mixing of the four trinucleotide systems is marked by the *dashed line*. *Fourth row*: dot plot of the MD simulation time (increasing from bottom to top, 0–200 ns) per snapshot. The follow-up run for G*m*^6^AC (“ext.”, *purple*) is stacked on top and differs in that the simulation time does not contain the time it already took in the initial sampling (“*red*”): thus, it is homogeneous for all replicas and spans only 0 to 100 ns. The horizontal gridlines give the zero time point per set. *Last row*: kinetic annotation based on the negative logarithm of the cut function assuming a three-state system. The cut function is directly proportional to the number of transitions in the original MD time progression between the sets of 10,000 snapshots to the left and to the right. High values indicate little sampling across the corresponding point, meaning that it belongs to a kinetic barrier region. The initial, equilibrated simulation snapshot of each replica is marked by a *colored circle* following the color code of the plot above. *m*^6^A, *N*6-methylated adenine; MD, molecular dynamics; PC, principal component; PIGS, Progress Index-Guided Sampling; SAPPHIRE, States And Pathways Projected at High Resolution.

The progress index (PI) (59) permits intuitive comprehension of the featurization by arranging snapshots so that self-similar sets form compact blocks along the x-axis. The resultant States And Pathways Projected at High Resolution (SAPPHIRE) plot (60) annotates these blocks geometrically and kinetically, and Fig. 8 reveals separate regions characterized by low (<6 Å) as well as high (>20 Å) distance between *m*^6^A and the aromatic cage. The first PC (16% of variance) is strongly correlated (*ρ* = 0.92) with this distance, which implies that the six remaining dimensions correspond to orthogonal structural features. Crucially, several structural substates for both high and low distance regions are resolved. This suggests that several metastable states are visited, whether on- or off-pathway, and successfully captured by the chosen representation.

Most of these metastable states are visited by all four systems, but differences in relative populations can be gleaned from the system-specific annotations of the PI and the entropy of mixing. The basin spanning PI values from 0 to 190,000 is the notable exception and is populated almost exclusively by snapshots derived from G*m*^6^AC. It contains all equilibrated starting configurations of that system while those for AAA, A*m*^6^AA, and GAC are located in the basins to the right. This suggests that the methylation of GAC introduces a specific disposition for RNA to assume a crystal-like configuration that is less accessible to both AAA and unmethylated GAC. Such a clear partitioning of the bound state is surprising, and we acknowledge that this might be a result of the parameterization in CHARMM where, in the unbound state, we observed changes on a more global scale compared to Amber (see above).

The basin corresponding to fully unbound configurations is located at *ca.* 780,000 < PI < 980,000 and separated by the dominant barrier on the PI. Because the initial PIGS run for G*m*^6^AC offered little in terms of unbinding, *q*_*MSM*_ guided us in how to pick starting configurations. The purple circles in Figure 8 (bottom) and the dot pattern for the simulation time of the original run demonstrate that PIGS was successful in diversification of the crystal structure for G*m*^6^AC albeit for very few events and that our strategy relying on clustering and *q*_*MSM*_ picks out these interesting points with very high fidelity. As expected, none of the starting snapshots for the second run (*i.e.*, the committor-based follow-up run of G*m*^6^AC) come from the original crystal-like basin on the PI. The simulation time trace for the second run indicates that, starting from these intermediate states, we managed to visit all metastable states, including unbound configurations, in a manner that resembles the coverage for the other three systems. We chose here, deliberately, a strategy to produce a system representation that results in a rich, structured distance spectrum rather than relying on an optimization framework (*cf.* Fig. 7) (61, 62, 63). This richness is reflected in a sample from the Euclidean distance spectrum in the transformed space of interatomic distances (Fig. 7) as well as an annotated 1D projection, the SAPPHIRE-plot (Fig. 8).

It is a possible limitation that we created all starting models from Protein Data Bank (PDB) entry 4R3I (20). Is it conceivable that some of the metastable states in Figure 8 are anticipated by experimentally determined structures? Similarly, is the binding site-focused nature of the metric we use above and below creating a misleading notion of similarity? To address both questions, we computed RMSD values for nonsymmetric heavy atoms that are not in side chains of D, E, K, or R (due to noise from frequent solvent exposure) from MD samples to a diverse set of experimental structures. We restricted ourselves to structures where the 129 contiguous residues from Asp354 to Leu482 were resolved with complete backbone heavy atoms. The set includes both *holo* (4R3I, models 1, 10, 20 from 2MTV (21), 6ZCN (chain B) (54), 7L4X, 7L4Y (64)), and *apo* forms (6ZD9 (54), either chain A or B), representing both NMR and X-ray structures and different categories of nucleic acid ligands. From our simulation data, we then identified the 100 snapshots most similar to each experimental structure. The choice of 100 snapshots is a compromise between having both contrast and robustness in the analysis.

Table 1 makes three important points. First, the snapshots most similar to all *holo* structures cluster in the basins where the central RNA base is still in the binding site, in particular the one where the equilibrated starting snapshots for GAC/AAA are found. They are all already seen during early time points. This means that the diversity these structures encode is confined to the bound state, and only a few ns of MD sampling are sufficient to cover this space. Second, we previously demonstrated two alternate conformations of the binding loop in the two chains of 6ZD9 (Met438 in/out) (54), and the one clearly incompatible with RNA binding (Met438 in, chain B) does in fact overwhelmingly map to the areas of the PI where the base is no longer in direct contact with Trp428 of the aromatic cage. Moreover, the fact that the minimum distance snapshots to both *apo* structures are obtained at much later simulation times than those to *holo* ones is consistent with the idea that *apo* and *holo* states are kinetically distinct, despite their high similarity experimentally. It is remarkable that this can be picked up even with a very broad metric as chosen here: the heavy-atom RMSD across 129 residues. Third, experimental modalities do play a role: both the NMR models from 2MTV and the crystal structure 7L4X, which is bound to (partially) double-stranded DNA, have structural features that make them similar to a wider range of conformational states than, *e.g*., 4R3I or 6ZCN.

**Table 1:** Comparison to experimental structures

Annotation	G*m*^6^AC	GAC/AAA	A*m*^6^AA	On-path	Fully unbound	*m*^6^A unbound	‹RMSD› (Å)	‹Time› (ns)
Progress index (×10^−6^)	*< 0.2*	*0.2–0.34*	*0.34–0.43*	*0.43–0.57*	*0.78–0.98*	*Other*		
4R3I (RNA)	0	93	7	0	0	0	0.82	0.9
NMR #1 (RNA)	10	44	14	29	0	3	1.19	15.6
NMR #10 (RNA)	7	44	16	24	0	9	1.22	11.5
NMR #20 (RNA)	8	60	9	21	0	2	1.17	14.3
6ZCN:B (*m*^6^A)	0	97	3	0	0	0	0.84	0.9
7L4X (DNA)	17	39	21	20	1	2	0.90	22.0
7L4Y (DNA)	1	93	0	6	0	0	0.83	5.1
6ZD9:A (none)	5	30	19	33	1	12	0.98	42.7
6ZD9:B (none)	6	5	7	7	31	44	1.32	57.9

Columns 2 to 7 list where the 100 closest snapshots to a given structure (column 1) can be found in Figure 8. The class of ligand in the experimental structure is given in parentheses. All NMR models are from 2MTV. The annotation (row 1) refers to the interpretation derived from the SAPPHIRE plot (Fig. 8), see text. “Time” refers to the simulation time within the single replica, which for the follow-up run does not include the time sampled in the original run (up to 200 ns). RMSD values are based on heavy atoms in 129 contiguous residues. They are slightly higher (both minimum and the average shown) for NMR structures than for other *holo* structures, presumably due to the differing experimental methodology. The averages are across the 100 closest snapshots.

In summary, the unbinding of the RNA trinucleotides, AAA, A*m*^6^AA, GAC, and G*m*^6^AC, is readily sampled by PIGS. In a space defined by a rich set of interatomic distances, unbinding trajectories visit states that are shared across systems. The committor-based reseeding, making further use of both existing trajectory data for G*m*^6^AC and control simulations with AAA, A*m*^6^AA, and GAC, dramatically improved the sampling of unbinding events for G*m*^6^AC. The total sampling time amounts to 4 × 64 × 200 ns (starting from the crystal structure) plus 64 × 100 ns (starting from intermediate committor values). From a simple analysis of counting unbinding events for AAA, we conservatively estimated a speed-up of roughly one order of magnitude with PIGS compared to conventional sampling (Fig. 5). It is thus reasonable to conclude that this strategy enables observation of processes occurring on a much longer timescale than that provided by the longest sampling time per replica (200–300 ns) and closer to the cumulative time of (at most) 19*μ*s per system. We investigate the timescales of unbinding of the four nucleotides from YTHDC1 as well as the shared (or unique) states in more detail in the following section.

### Unbinding of G*m*^6^AC occurs on the *μ*s timescale in high salt

MSMs synergize well with the adaptive nature of PIGS. Once the system transitions from an initial state to a new configuration, that configuration is recognized as nonredundant and will thus be a candidate for replacing copies offering only redundant information. While this does not increase the effective number of independently observed events, the reseeding permits more observations resulting from stochastically evolving the system starting in state *i*. Therefore, some of the conditional probabilities $P\left(x_{t+\Delta t}=i|x_{t}=j\right)$, which lie at the core of the MSM, can be estimated with higher accuracy compared to conventional sampling. The increase in accuracy is found in particular in transition regions, where good estimates of $P\left(x_{t+\Delta t}=i|x_{t}=j\right)$ are both crucial for capturing the system’s dynamics but also difficult to obtain with conventional sampling due to the inherently low population.

MSMs are estimated for each of the four systems separately but based on a joint clustering. This enforces that the same resolution be applied to each system. Additionally, states can be matched exactly, even though, unsurprisingly, not all states are visited by every system. Out of 1954 MSM clusters in total, 1013 clusters (containing 811,779 out of 1,151,616 snapshots) are shared by all four systems (Table S1). Furthermore, only 2% of the total sampling (508 clusters made up of 27,457 snapshots) is exclusive to one or two nucleotide systems. The clustering resolution and the lag time are among the principal parameter choices of MSMs; objectively correct values cannot be easily known or selected. Figure S11 suggests that the MSM’s main output used in this study, the committor, is rather robust over the tested range of parameter values. We have previously argued that implied timescales are not reliable diagnostic tools (65), and, also here, Fig. S12 offers no clear guidance. Given the low sensitivity of the committor, we deemed a model constructed with a lag time of 1 ns with the clustering resolution chosen to be 1954 clusters as appropriate. This is the finest resolution where each individual, trinucleotide-specific MSM results in a single connected component (a few singlet “clusters” notwithstanding).

For a kinetic description of the unbinding process(es) sampled in each of the systems, we turn to TPT, which posits that two sets of nodes (synonymous with clusters) must be declared boundary states, *U* and *B*. As explained in “Supplementary Methods, Transition path theory (TPT),” this represents a conceptually intuitive imposition of two-state logic, akin to the analyses of many experimental data, onto the MSM, which in turn is a discretized model of the MD trajectories. The imposition is most appropriate if there is strong separation of timescales caused by a single barrier separating two end states exhibiting fast, internal relaxation. Here, we define *U* as the unbound state. This state encompasses all clusters for which the centroid of said cluster has a distance larger than 25 Å from adenine’s *N*6 atom to the aromatic cage. State *B* is defined as the set of nodes that contain any of the 4 × 64 starting snapshots (see Table S2). This lumping of clusters into larger states can introduce shortcuts into the network, which would compromise the estimation of timescales. For example, by virtue of defining a homogeneous unbound state purely based on distance from the binding site, we create a state that contains the RNA in very different positions relative to the domain, just all distant. This is imposing the assumption regarding internal relaxation above, here, that diffusing in solution does not make a relevant contribution toward the on-rate.

Kemény’s constant is a measure of the expected (weighted, average) time to travel between any two states in a Markovian network. The values for Kemény’s constant (66) reported in Table 2 indicate that our coarse-graining does not create drastic shortcuts in any of the four system-specific networks. The mean first-passage time to pass between any pair of nodes is reduced to 80% at most, compared to the network at the finest resolution we consider, and this is almost entirely due to the lumping for *U*. Conversely, defining state *B* as described above alters the global dynamical properties of the network only marginally. The definition of boundaries *U* and *B* has effects on the connectivity of MSM modeling that is consistent for all four systems, G*m*^6^AC, GAC, AAA, and A*m*^6^AA. We acknowledge that this is purely in relation to the original clustering and not an assessment in more global terms.

**Table 2:** Kemény constant in units of ns

Lumping	G*m*^*6*^*AC*	GAC	A*m*^*6*^*AA*	AAA
Full res.	2637 (100%)	2583 (100%)	2792 (100%)	2491 (100%)
*B*	2621 (99%)	2557 (99%)	2774 (99%)	2469 (99%)
*U*	2122 (80%)	2193 (85%)	2342 (84%)	2069 (83%)
*B* and *U*	2106 (80%)	2167 (84%)	2324 (83%)	2046 (82%)

The different rows correspond to networks where the bound state (*B*), the unbound state (*U*), or both have been lumped based on the criteria given in the text. The data for G*m*^6^AC are based on MSMs that include the additional sampling generated from intermediate values of *q*.

The lack of a kinetically homogeneous “unbound” state is a typical feature of molecular systems (58), and, in this case, the heterogeneity is deliberately masked by a data transformation. A sigmoidal transformation of the selected intermolecular and intramolecular distances artificially coerces large values to be treated as homogeneous (invariant). We note that such transformations only permit a more compact coarse-graining but do not address the issues of shortcuts and glossing over slow modes in equilibrating conformations within states when these states are intrinsically heterogeneous. This applies both to the original clustering and the lumping step. Here, we also defined a (somewhat arbitrary) distance threshold for the lumping of *U*. Tighter definitions, which tend to remove some of the shortcuts, will inevitably result in “intermediate” states that, intuitively, should be unbound. It is desirable but difficult to avoid such definitions altogether. As an alternative, we here check for the robustness of either specific observables or global network properties such as Kemény’s constant.

With two boundary states defined, TPT permits calculation of the committor *q*_*MSM*_, which in the following differs from the analogous quantity referenced in Fig. S7 by the inclusion of all PIGS trajectories, including the reseeded PIGS ensemble of G*m*^6^AC.

**Figure 9:**
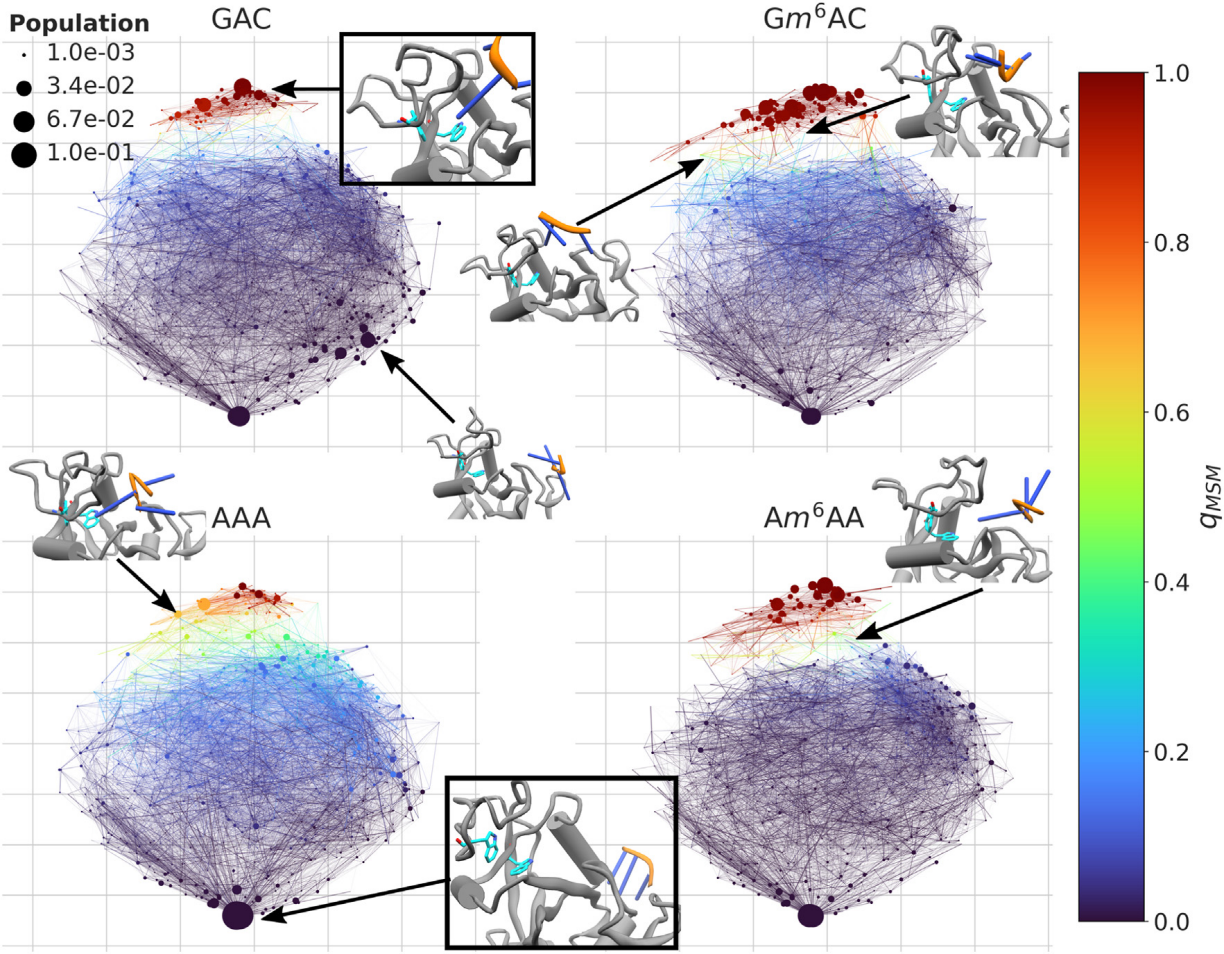
**Multiple pathways of (un)binding of m6A-RNA from YTHDC1.** The networks represent the projection of the joint clustering for each of the four separate trinucleotide systems. The two-dimensional embedding is determined by MDS of the centroids’ pairwise distances (*cf.*Fig. 7). The states are represented by *circles*, which are colored according to their system-specific *q*_*MSM*_ values and scaled by their stationary weight. The edges’ widths represent the transition probabilities, and their color encodes the average *q*_*MSM*_ of the two states they connect. For a small selection of states, a Cartoon representation of the cluster’s centroid is shown. In these, the protein is in *gray*, the RNA is in *orange* (backbone) and *blue* (bases), and the aromatic cage is in *cyan*. Two centroid configurations of clusters belonging to U and B, respectively, are framed. *m*^6^A, *N*6-methylated adenine; MDS, multidimensional scaling; MSM, Markov state model.

The matched states including dynamical information obtained by TPT are leveraged for the 2D representation shown in Fig. 9. The position of each state is determined by using multidimensional scaling, which embeds the pairwise distances of centroids in the familiar representation described in “Experimental procedures: System featurization and SAPPHIRE plot” in a 2D space. The size of each node is scaled by the stationary probability of the state it represents. Additionally, the committor of each state is annotated in Fig. 9 by a color code.

From the mutual similarity of the graph visualizations, it emerges that most major nodes are shared between systems, which is consistent with Figure 8. Native-like nodes (red, North sector of the multidimensional scaling-embedded network) and the same major unbound node (South sector) exhibit consistently high weight. System-specific states of substantial weight are found only in few regions of the network projections. For instance, G*m*^6^AC is characterized by several bound states that are not found, or only found with negligible weight, for the negative controls. In the centroids of these states, the RNA’s backbone is twisted and detached from its crystal-like position on the protein surface (see insets on Fig. 9).

In contrast, the major unbound node and bound states have, respectively, the highest and lowest statistical weights in AAA compared to the other systems. This is consistent with its role as the strongest negative control. Some of the states that are structurally most similar to the crystal are marked by an intermediate committor value, which might be related to relaxation of the protein itself. Moreover, several states with high statistical weight, which are, however, distinct from the major unbound node, are visited in GAC (South East sector). These states are the result of the formation of various encounter complexes by GAC. The backbone interacts with the positively charged patch of protein residues in the general vicinity of the canonical binding site, but in a variety of configurations with the adenine base distant from the binding pocket.

The committor values (color annotation) and low populations suggest that states in the North West sector might be unproductive for A*m*^6^AA, which contrasts G*m*^6^AC. The insets in Figure 9 suggest that transitions *via* this region of the embedded network correspond to unbinding accompanied by a displaced RNA backbone and a closed protein loop. In A*m*^6^AA, unbinding seems to occur preferentially with a wide-open loop, such that the RNA’s backbone can largely retain the crystal configuration. One might therefore conclude that the altered RNA sequence precludes the trinucleotide from passaging through these states, *i.e.*, it is a pathway that is specific to the RNA’s sequence context and that depends, in a nontrivial manner, on both bound and unbound states.

As alluded to before, it is a caveat that such observations may be FF-specific. Indeed, the RNA, methylated or not, changes its conformational preferences depending on the FF. It is therefore unclear whether this property of the simulation translates to the system *in cellulo*. Model errors aside, TPT is a framework that supports the identification of dominant pathways in principle. While we attempted to pinpoint sequence- or methylation-specific dominant pathways, this investigation proved inconclusive, see “Supplementary Methods, Transition path theory (TPT).” All four networks in Figure 9 display a similar dense connectivity and hint at many pathways contributing to the observed net process. This multitude of pathways is consistent with an association mechanism driven by electrostatic steering between polyions, similar to what we observed before (67). The analysis above can be supplemented quantitatively by deriving rate constants from the committor along with the MSMs’ transition matrices following standard TPT (68). From Table 3, it emerges that the association rate constant, *k*_*on*_, is different for G*m*^6^AC compared to the negative controls. When either sequence or methylation state are not canonical, *k*_*on*_ decreases from 0.3 mM^−1^*μ*s^−1^ by roughly one order of magnitude.

**Table 3:** Rate constants and dissociation constant derived from TPT analysis

System	*k*_*on*_[M^−1^μs^−1^]	*k*_*off*_ [μs^−1^]	*K*_*D*_ [mM]
AAA	70 (50–100)	10.8 (5.75–16.04)	150 (59–331)
A*m*^6^AA	40 (20–90)	0.73 (0.42–1.34)	17 (5–88)
GAC	50 (20–80)	2.05 (1.02–4.05)	44 (12–229)
G*m*^6^AC	300 (120–490)	0.26 (0.18–0.45)	0.87 (0.42–4.58)

Values in parentheses are empirical 90% confidence intervals from 20 repeats with subsampled transition matrices.

The dissociation rate constant, *k*_*off*_, provides contrast between all four systems and therefore delineates AAA, A*m*^6^AA, and GAC. Whereas the removal of the methylation of *N*6 is marked by a tenfold acceleration of unbinding, a changing of the consensus sequence from G*m*^6^AC to A*m*^6^AA (retaining the methylation) is less drastic; the dissociation rate constant only triples from 0.26 *μs*^−1^ to 0.73 *μs*^−1^. For the unmethylated trinucleotide AAA, *k*_*off*_ is increased by more than what one would expect from independent effects of sequence and methylation. The individual modifications to G*m*^6^AC account for a 2.8-fold (altering the sequence) and 7.9-fold (removing the methyl) change, respectively. The loss of both features jointly is characterized by a dissociation rate constant of 10.8 *μs*^−1^, which is a 42-fold acceleration compared to G*m*^6^AC and indicates a roughly twofold, cooperative increase.

The resultant dissociation constants, *K*_*D*_, naturally reveal that AAA is least stably bound with a high-mM *K*_*D*_. A*m*^6^AA and GAC are each one order of magnitude more stable, and combining the two modifications results in a high-*μ*M dissociation constant for G*m*^6^AC.

Naturally, these estimates carry various errors, including statistical errors that derive from the fact that the transition counts are inevitably undersampled (69). There is by now a large number of heterogeneous approaches to deal with this type of uncertainty that often presuppose and vary different elements, such as the discretization itself. Here, we focus only on the count matrix that we augment, following prior work (65), with geometrically informed pseudocounts to improve robustness. We also subsample it by removing 1% of counts at random to produce the 90% confidence intervals in Table 3 (from 20 repeats). The confidence intervals mirror the trends of the numbers obtained for the full networks, and allow the conclusion that the differences in *K*_*D*_ of G*m*^6^AC *vs* A*m*^6^AA/GAC *vs* AAA are significant, as are all differences in *k*_*off*_.

## Discussion

We have used an adaptive sampling strategy called PIGS (55) for the examination of oligo-RNA unbinding from a protein, a process that occurs on timescales of *μ*s or more. In previously published accounts as well as our own simulation attempts, *m*^6^A embedded in the DRACH consensus sequence binds too tightly to YTHDC1 for unbinding to be tractable with conventional MD (37, 54). We tackle this issue by combining adaptive sampling with a simplification of the simulation system. The pentanucleotide motif DRACH is reduced to the central trinucleotide segment, focusing on the most conserved positions, RAC. The combination of adaptive sampling and a simplified trinucleotide model is additive in accelerating unbinding with respect to canonical sampling and the pentanucleotide system. We relied on control simulations, where the consensus G*m*^6^AC is replaced by A*m*^6^AA, GAC, and AAA, to contextualize the results for G*m*^6^AC as well as to inform the sampler for improved exploration. Furthermore, the four trinucleotides allow dissection of the sequence specificity from the effect of the methylation. We find that both properties affect the unbinding in a coupled fashion, with the methylation of *N*6 having a somewhat larger impact, as expected.

In reality, the DRACH motif will be embedded in a much longer RNA sequence, usually mRNA, when bound to the YTHDC1 domain (18). This means that the unbound state of RNA is readily modulated by hybridization or nonspecific association. This can occur both intramolecularly (53) and intermolecularly (42), and the resultant complexity provides mechanisms for highly indirect alterations of the binding stemming from distal sites (whether protein or RNA), one particularly stunning example being the formation of biomolecular condensates containing both RNA and YTHDC1 (70). This wider view of unbound state effects is undoubtedly essential for understanding the behavior of the cell and explaining some *in vitro* results (see below), but the underlying processes, hybridization, and folding, are out of reach for routine, atomistic simulations and challenging to capture even in coarse-grained models (71), at least without sacrificing significant spatial resolution (72). Our results of the pentanucleotides in water primarily provide a hypothesis for why one might see differences within the stylized settings we have adopted here for the simulations of protein-RNA complexes.

Our RNA-only simulations exhibit nontrivial differences for the two FFs, CHARMM, and Amber. RNA and DNA FFs are mostly parameterized, tested, and refined with folded states in mind, which can lead to residual biases for single-stranded segments, such as excessive base stacking or misleading puckering preferences (44, 73). They are also in constant flux (36), with numerous, problem-specific adjustments being proposed (25, 74), while focusing on tractable systems, mostly tetraloops (26). In the unpaired state, the dihedral angle around *m*^6^ should favor the *anti*-conformation with the methyl pointing away from the 5-ring, a preference that is inverted in the paired state (42, 75). On the limited time scales available, we observe isomerization from the initial *syn*-configuration in CHARMM but not in Amber (75, 76). A tendency toward lower free energy barriers in CHARMM than in Amber emerges in general (Fig. 2, top panel), suggesting that configurational fluctuations, which are likely required to facilitate unbinding, are accessed more easily. In the context of our simulations of the complex, it is exactly these spontaneous fluctuations that are readily amplified by PIGS: the algorithm exploits them to promote exploration in an adaptive manner (55).

While comparative studies of FFs are fundamental at the community level to understand the limitations of different models, there are almost always two additional factors to consider. Can the models be sampled? Are there additional species creating heterotypic interactions (protein, water, and ions)? Even basic thermodynamic signatures of biomolecular processes can depend on the water model more so than on the FF (34). Here, our guiding principle in choosing CHARMM was the need to obtain sufficient sampling. In Tucker *et al.* (77), four out of six protein-RNA complexes were virtually rigid on the 50 μs timescale using an Amber variant. Irrespective of how reasonable this is, it is impractical for sampling larger-scale transitions, and our tests with Amber resembled this scenario.

From the above, it is clear that both sequence and FF can thus incur changes to the observable on- and off-rates. That said, the fact that there is sequence specificity is not obvious from a structural point of view as both surrounding bases point outward, Figure 1, and water-mediated effects have been proposed as a possible mechanism (20, 37). It appears that our approach coupled to the CHARMM FF describes these challenging systems accurately enough for drawing insightful conclusions, which we infer both structurally and thermodynamically. The data in Table 1 highlight that the unbound state in our simulations, which all started from the same *holo* protein, can closely resemble an *apo* crystal structure with the binding site blocked. This is convincing evidence that the states discovered by the combination of FF and sampling paradigm are realistic. Moreover, the accuracy of the resultant ranking of binding affinities as seen in Table 3 for the complex of YTHDC1 and trinucleotides summarizes the trends extractable from various isothermal titration calorimetry (ITC) experiments well. For clarity, our study provides no evidence that Amber does not describe the system accurately. We anticipate that the strategies presented here will be particularly useful while FFs are further optimized for heterogeneous systems, which is an active field of research (7, 78, 79, 80).

The acceleration conferred by PIGS generally depends on the balance of entropic and enthalpic barriers to be overcome, which is system-specific. In the present case, the removal of two of the negative charges on the backbone, which sacrifices sequence-unspecific interactions in favor of, putatively, a substantial reduction of the enthalpic contributions to binding, is likely to have facilitated the spontaneous unbinding observed here. We note that ITC data universally suggest favorable enthalpies for binding RNA to YTHDC1, but that these vary much more substantially than free energies do (21, 81). In our setup, the high concentration of monovalent ions was primarily intended to reduce the interaction of the RNA phosphate backbone with basic protein sidechain residues further. That said, the counterion concentration may specifically impact the RNA’s structure (82) and/or modulate intermolecular interactions in a more general sense (8). Importantly, we make no explicit assumptions about reaction coordinates or the nature of the underlying free energy surface. This is an advantage because these system properties are usually difficult to anticipate, especially for flexible macromolecules.

The adaptive nature of PIGS permits a seamless analysis using MSMs (65), a framework that natively handles the tree-like connectivity of the trajectory ensemble. We supplement the empirical verification that the committor, the main output of our analysis, is robust with respect to lag time and clustering choices, with an explicit statistical assessment based on subsampling the count matrix. These intrinsic controls do not eliminate the challenge of assessing the fidelity of MSMs in modeling the data (58, 65). Moreover, we found that it is infeasible to determine dominant pathways of (un-)binding: neither a decomposition of the reactive flux nor the clustering of productive trajectories of random walkers proved conclusive. This finding is consistent with the lack of significantly populated routes on the 2-dimensional projections of the free-energy surfaces in Figure 9. It appears that flexible molecules (*i.e.*, the protein loop and the RNA itself) characterized by polyvalent interactions lead to highly stochastic, noisy processes, which are not readily captured by intuitive and visually interpretable pathway representations. We note that this might be a functional prototype: previously, we discovered that the binding of a peptide to its cognate PDZ protein domain proceeds through multiple pathways involving nonspecific salt bridges (67). This hints at a shared origin: evolution might favor versatility and fast rates in these interactions to facilitate hyperfine regulation of cellular processes on short enough timescales.

Ranked or relative affinities are, as opposed to their absolute counterparts, safer to relate to experiment (39), not least because the solution conditions here, and in MD in general, are highly stylized. While kinetic studies of RNA-protein interactions are common (83), we are unaware of measured rate constants for short oligonucleotides binding to YTHDC1, which might be because the process is, for most methods, prohibitively fast (the on-rates in Table 3 are near the diffusion-limited regime, ∼10^8^ M^−1^s^−1^). In terms of relative, thermodynamic effects, our results are consistent with data from ITC experiments. The cognate trinucleotide sequence was found to have an affinity of 28 *μM* (20). In the same work, the change from GG*m*^6^ACU to GA*m*^6^ACU either in the pentanucleotide or in longer chains brought about a 6 to 8-fold loss in affinity while the drop from GG*m*^6^ACU to GG*m*^6^AAU was 3-fold. This suggests a reduction in affinity by a factor of 15 to 25 in going from G*m*^6^AA to A*m*^6^AA, which compares very well with Table 3. Other experimental results point in a similar direction: Xu *et al.* report a 5-fold loss for changing G*m*^6^AC to A*m*^6^AC in a longer chain (84). Li *et al.* find that pruning D and H from DRACH leads to a 7-fold and 20-fold reduction, respectively, compared to the 0.5 *μM* for GG*m*^6^ACU (19). If these effects were independent, it would predict an affinity of ca. 70 *μM* for the cognate trinucleotide. From this, we conclude that the *K*_*D*_ values in Table 3 capture the right trends regarding sequence context; the affinity is too low by a factor of 10 to 25, however. It is worth noting that the specific modalities of the ITC experiments also contribute differences not explained by statistics: for example, the affinity of the pentanucleotide to YTHDC1 is reported alternatively as 0.5 ± 0.0 *μM* (19) or 2.0 ± 0.1 *μM* (20). Moreover, ITC is usually performed in lower salt concentrations: the trinucleotide affinity of 28 *μM* was obtained in 150 mM NaCl and 30 mM Tris (20), which is a more than 5-fold lower ionic strength than our simulations.

Regarding the specific effect of methylation, less data are available, but Theler *et al.* measured a 50-fold loss in affinity for YTHDC1 when demethylating UG*m*^6^ACAC (21), which is exactly what we find in Table 3 for the cognate trinucleotide sequence. Generally speaking, the RNA sequence matters also for distal positions, in particular for high-affinity binding: for example, GAACCGG*m*^6^ACUGUCUUA (20) and CGCGG*m*^6^ACUCUG (81) are both nM binders, but the latter binds tighter: it is clear that these details are beyond the scope of our study. The existence of binders with very high affinities, in particular for DC1 relative to other YTH domains, and the possibility of complex unbound-state effects as discussed above place a caveat on our results for the diversity of pathways. It appears that the engineering toward versatility we propose above can only hold for RNA that behaves like unstructured, single-stranded RNA near the recognition motif.

Finally, we discuss possible reasons for why quantitative predictions might suffer. First, it is a limitation that several molecular interactions of the system under scrutiny are difficult to be treated accurately. Despite recent advances, the quantitatively correct modeling of disorder remains a challenge in MD (28, 35, 85, 86, 87), which is noteworthy because the flexible loop covering the aromatic cage seems to be heavily implied in (un-)binding processes. Similarly, classical FFs have no explicit treatment of methyl-*π* interactions in the cage: they approximate base stacking by a mix of standard nonbonded potentials and preorganization (44, 73). Second, the system might be too stylized in terms of solution conditions: we, like most studies, use single copy numbers for the polymers and disregard the binding of divalent, usually Mg^2+^, ions to the phosphate backbone (88). Third, as a polyelectrolyte, any form of RNA in water is difficult to model, and it is not surprising that RNA parameters have seen various revisions over the years (25, 26, 27, 28). Thus, describing the interactions of unstructured RNA, ions, proteins, and a water model is arguably a formidable challenge with current FFs (32, 33). In some applications, the issues mentioned above have lead researchers to devise *ad hoc* potentials to prevent undesired behavior of RNA-protein-complexes (12, 37, 89), which we do not consider here.

Even a maximally reductionist approach to the composition of the system under study, *i.e.* a single copy of oligo-RNA and protein immersed in a bath of explicit water containing ions at fixed concentrations, is thus on the fringe of what is reasonable to treat with current classical FFs, and the use of a classical model is a concession itself. While the *K*_*D*_ value for G*m*^6^AC in the high micromolar range is likely off by an order of magnitude, it is therefore encouraging that a relative ranking of affinities in good agreement with experiment is achieved (19, 20, 21, 84).

## Experimental procedures

### Pentanucleotides in water

The CHARMM36m FF (51, 87, 90) (GROMACS-port from March 2019) as well as a FF from the Amber family were used to perform simulations of 5′-GGACU-3′ and 5′-GG*m*^6^ACU-3′ in water. In addition, 150 mM NaCl was added in excess. For CHARMM36m, the structure was solvated in its recommended modified version of TIP3P water. Additional details can be found in Tables S3 and S4. AmberTools20 (91) was used to prepare models using the ff99bsc0-*χ*OL3 (92, 93) parameter set including the Steinbrecher–Case modification of the Lennard–Jones radius for backbone phosphates (94) for description of the nucleotides. *m*^6^A was described using published parameters (37), and the OPC water model was used (95). Li/Merz ion parameters (96) described the ions, and we used AmberTools20 to convert the resultant topology to GROMACS-compatible files.

The system was prepared using both FFs in 16 replicates, which were propagated until a simulation time of 200 ns was reached. Six of the initial structures originate from the crystal configuration in PDB 4R3I (20) with different initial velocities, and 10 were uniformly sampled from a 50 ns simulation of the crystal structure at 380K (Fig. S13).

The 31 backbone dihedral angles of the pentanucleotide were projected onto 10 PCs (40), preserving 83% of the variance. A tree-based clustering (97) served to partition trajectories into 37 states. The smallest cluster radius was set to 2.159° with respect to Euclidean distance in PC-space. Base stacking and pucker configurations were calculated using the Barnaba software (https://github.com/srnas/barnaba) (98).

### PIGS simulations of unbinding

Simulations using the CHARMM36m FF (March 2019) were launched with each of GAC, G*m*^6^AC, AAA, and A*m*^6^AA initially bound to YTHDC1 as derived from PDB entry 4R3I. The complex was enclosed in a cubic box of 70.7 Å^3^ filled with TIP3P water and 1M excess NaCl. Configurations were equilibrated at 300K and 1 bar for 1 ns. Production runs were started from the snapshot closest to the average volume. The box volume and the average temperature of 300K were held fixed. For the latter, the velocity rescaling (99) thermostat with a coupling time of either 10 ps (A*m*^6^AA, an unintended deviation discovered later) or 100 ps was used. The long coupling time was to minimize the quenching of spontaneous fluctuations. Due to the conservative integration settings (see also Table S4), this had little effect on how well temperature could be maintained (Table S5). Trajectory input files were prepared and propagated using GROMACS 2020.3 (100).

The reseeding heuristic at the core of PIGS was calculated with CAMPARIv4 (http://campari.sourceforge.net). It is defined in terms of 32 dihedral angles (Table S6). Subsequently, 18 of those angles characterize the trinucleotide with all available nonredundant sugar, phosphate-backbone and glycosidic angles taken into account, while the remaining 14 are polypeptide *ϕ*- and *ψ*-angles. After 50 ns of PIGS simulations of the different trinucleotide-DC1 complexes, the diversification of features up to that point was evaluated. Since several nucleotide angles reached a near-uniform distribution, some phosphate backbone angles were pruned from the feature set. They included *ζ*- and *α*-angles for both the central and the 3′-nucleotide as well as the *γ*-angle of the central adenine.

In addition to removing those five dihedral angles characterizing the RNA configuration, additional protein angles were included in the feature set such that the revised set of features contained 13 RNA angles and the *ϕ*- and *ψ*-angles of 15 protein residues (30 protein angles total) surrounding the binding site. Both the newly added and the previously chosen protein features exhibited a low degree of diversification, which is in part expected as they are part of a folded polymer, posing much stronger constraints on conformational exploration, even for loop residues, than single-stranded RNA. The ensemble of trajectories of G*m*^6^AC (re-)started from clusters with a low committor-value used the revised feature set for the full 100 ns of PIGS.

For each of the four systems, 64 replicas were propagated for 200 ns. Reseeding of at most 32 replicas occurred every 100 ps.

### System featurization and SAPPHIRE plot

A differential cutoff map was constructed (Fig. S10, top panel). The shortest distance between any two heavy atoms between all residue pairs of the system was calculated. The distance was counted as a contact if the distance is shorter than 5 Å. Such a contact map was constructed separately for the first and the last 25 ns of the 200 ns of simulation time per replica. A residue pair was considered for featurization if the absolute difference in contact frequency between two residues exceeded 0.35. This resulted in 64 residue pairs (Fig. S10, bottom panel).

Several intraprotein residue pairs were discarded manually as they are related to, for example, the partial loss of helicity at the termini. The final selection of residues is shown in the top panel of Figure 7. For each protein residue, both the C*α* and the most distant side chain heavy atoms were selected for the construction of pairwise distances. For each of the three RNA residues, three atoms were chosen: C4′ and O3′ for all residues, *N*9 for G and A, and *N1* for C.

Intraprotein and intermolecular residue pairs were restricted to pairs meeting the chosen cutoffs. All three RNA-RNA residue pairs were considered regardless of contact frequencies. By exhaustively forming atom pairs (four for intraprotein, nine for intra-RNA, and six for intermolecular residue pairs) for all selected residue pairs, the unbinding of RNA from YTHDC1 was described in the end by a feature set of 262 interatomic distances.

These distances were first transformed by a sigmoidal transform, $\phi \left(x\right)=1-{\left(1+\exp\left(-\left(x-\chi \right)/\tau \right)\right)}^{-1}$, which is roughly linear around 15 Å and flattens out near 5 and 25 Å (Fig. 7, bottom). They are subsequently reduced to seven dimensions using PC analysis, which, for the single systems, preserves >60% of the total variance and 34% in the combined representation. We note that the reference (“ideal”) distributions differ for intramolecular and intermolecular contacts if we take molecular topology as given: the former are bound by sequence spacing whereas the latter are bound by the simulation container.

The PI (59, 60) arranges all 1,151,616 snapshots so that self-similar snapshots are found in similar parts of the plot. This is based on a distance metric: here, the Euclidean distance in the feature space described above. The approximate PI (59) was calculated based on a tree-based clustering (97) with the minimal and coarsest cluster radii set to 0.2 and 3.5, respectively. The three outermost layers of leaves were folded inward onto their parent vertices (101). The kinetic annotation was calculated for a three-state model of 10,000 snapshots around the current PI. The entropy of the distribution of the annotation which system a snapshot originated from was computed in a rolling window of 2000 snapshots.

### MSM construction and rate constant calculation

The tree-based clustering was used for discretization of all four systems jointly with the transformed, interatomic distances serving as the feature space. On these joint data, the clustering resulted in 1954 clusters. An MSM was constructed for each of the four systems with a lag time of 1 ns using a sliding window to count transitions (Fig. S12). A prior transition count was added according to the structural similarity of the involved clusters, as suggested in prior work (65) (bin width: 0.002). No symmetry of the count matrix was enforced.

TPT offers a framework for the calculation of transition kinetics between two boundary states separated by intermediate states. TPT was thus used for the calculation of (un-)binding rates. State *B* was defined as the union of clusters containing the starting snapshots in each system. State *U* included all clusters for which the centroid snapshot was characterized by a distance between the aromatic cage and *N*6 of more than 25 Å. Quantification of the apparent invariance of the Kemény constant was used to verify that this coarse-graining did not introduce drastic shortcuts into the network, and that the definition of boundaries has comparable effects on the connectivity of all four networks.

The productive flux $f_{ij}^{+}$ between pairs of nodes *i* and *j* is calculated from the transition matrix $T_{ij}=P\left(x_{t}=j|x_{t-\Delta t}=i\right)$, the stationary probability $\pi_{i}$ and the plus- and minus-committors, $q_{i}^{+},q_{j}^{-}$, as

(1) $$f_{ij}=\pi_{i}T_{ij}q_{i}^{-}q_{j}^{+}$$

(2) $$f_{ij}^{+}=\max\left\{0,f_{ij}-f_{ji}\right\}$$

(3) $$F_{BU}=\sum_{i\in B}\sum_{j\notin B}f_{ij}^{+}=\sum_{i\in B}\sum_{j\notin B}\pi_{i}T_{ij}q_{j}^{+}=\sum_{i\notin U}\sum_{j\in U}\pi_{i}T_{ij}q_{i}^{-}$$

*F*_*BU*_ denotes the total current between the set of source nodes *B* and a set of target nodes *U*, and it must be the same as both the net outgoing flux (from *B*, $q_{i}^{-}=1$) and the net incoming flux (into *U*, $q_{j}^{+}=1$). It can be interpreted as the fraction of trajectories that are reactive $F_{BU}=\lim_{t\to \infty }N_{T}/T$. The forward and backward rates $\nu_{BU},\nu_{UB}$ are then given by:

(4) $$\nu_{BU}=\frac{F_{BU}}{\tau \sum_{i}^{N}\pi_{i}q_{i}^{-}}$$

(5) $$\nu_{UB}=\frac{F_{UB}}{\tau \sum_{i}^{N}\pi_{i}\left(1-q_{i}^{-}\right)}$$

where *τ* is the lag time of the MSM. Bulk rate laws consistent with the law of mass action express reaction rates in terms of rate constants and effective concentrations of free reactants, here [*L*] and [*R*], and the complex [*LR*], where *L* denotes the ligand, *viz.* the different trinucleotides, and *R* its receptor, YTHDC1. Our simulations contain a single copy each, so [*L*] = [*R*]. Mass action prescribes that:

(6) $$\nu_{UB}=\frac{d\left[LR\right]}{dt}=\left[L\right]\left[R\right]k_{on}-\left[LR\right]k_{off}$$

(7) $$\nu_{BU}=\frac{d\left[L\right]}{dt}=-\left[L\right]\left[R\right]k_{on}+\left[LR\right]k_{off}$$

By operating on the modified flux network, $f_{ij}^{+}$, that disallows backward flux due to the max-operation, the rate constants can be expressed as:

(8) $$k_{on}=\frac{\nu_{UB}}{\left[L\right]\left[R\right]}$$

(9) $$k_{off}=\frac{\nu_{BU}}{\left[LR\right]}$$

The fraction of the intact complex is calculated according to the combined stationary probability of states with a committor value greater than 0.5. This is the most intuitive definition to provide a quantitative mapping to a two-state system, but we note that it does not and (should not) entail a clear geometric annotation. This is different from many experiments that rely on probes that have specific structural origins (like the quenching of tryptophan fluorescence), and on baselines to map data to two-state models. In our case, the effective concentration of the complex is:

(10) $$\left[LR\right]=C\sum_{i}\pi_{i}1_{q_{i}^{+}>0.5}$$

where *C* denotes the total concentration of solute protein in the ligand box. The resulting ratio of rate constants reported in Table 3 is robust over a wide range of cutoff-values (Fig. S14).

## Data availability

Trajectory data are available through direct-access links to a specialized hosting platform where they can be downloaded (button ”Download”) but also visualized in the browser. The link for the trajectories of YTHDC1 and 5′-G*m*^6^AC-3′ in water is available through the following URL: https://acgui.bioc.uzh.ch/acgui/?sql_db=acgui_trajectories&sql_id=DC1_Gm6AC_CHARMM_PIGS&sql_load=1.

To directly access the other complex data, the Gm6AC in DC1_Gm6AC_CHARMM_PIGS is to be replaced with GAC, AAA, or Am6AA. To access the pentanucleotide-in-water data, use identifiers GGACU_Amber and GGm6ACU_Amber (for the Amber force field), and Gm6ACU_CHARMM36m or Gm6ACU_CHARMM36m for CHARMM. These values replace DC1_Gm6AC_CHARMM_PIGS in the above link. Alternatively, in-site navigation can be used.

## Supporting Information

Supplementary data (Supplementary Methods, Figs. S1–S14, Tables S1–S6) are available: they are uploaded as a separate PDF.

## Conflict of interest

The authors declare that they have no conflicts of interest with the contents of this article.
