# Supplementary material for: On the specificity of the recognition of m6A-RNA by YTH reader domains
Source: J Biol Chem. 2024 Nov 17;300(12):107998. doi: 10.1016/j.jbc.2024.107998 (PMC11699332; doi:10.1016/j.jbc.2024.107998)
Supplement: Supporting information [file mmc1.pdf]

SUPPLEMENTARY INFORMATION FOR:

On the specificity of the recognition of m6A-RNA by YTH reader domains

Julian Widmer, Andreas Vitalis,* and Amedeo Caflisch

Department of Biochemistry, University of Zurich, Winterthurerstr. 190, 8057 Zurich, Switzerland

E-mail: a.vitalis@bioc.uzh.ch

Supplementary Methods

Parameterization of m^6A

As a nonstandard nucleobase, m^6A is not part of the “core” set of force field (FF) parameters in either CHARMM or Amber. This is important because parameters of standard entities often have unique requirements towards consistency (analogous entities should have similar parameters), and are refined over many iteration cycles. When amending a FF, it is standard practice to follow its basic parameterization paradigm as much as possible, but the level of refinement and applied tweaks will inevitably have to deviate. Here, we resorted to two such parameterizations found in the literature. Since m^6A differs only marginally from adenine, it is logical to expect consistency with adenine parameters as the main guiding principle. While these are shared for Lennard-Jones and bonded parameters in Amber,[1] this does not hold for partial charges. In CHARMM, it is worth noting that the parameterization of m^6A in principle affects all parameters of the base (but not of the sugar or phosphate, where they are again shared, as expected).[2]

How similar are the two sets of partial charges to each other and to the ones in their parent force fields? Across the shared scaffold of the base (13 atoms), we find unexpected differences: for example, the Amber charges for the unmodified base are closer to those of CHARMM (RMSD: 0.12, Pearson: 0.97) than to those of m^6A in Amber (RMSD: 0.15, Pearson: 0.96). The total polarization of the atoms C8 and H8 by the two surrounding nitrogen atoms, N7 and N9, is strongest in CHARMM m^6A (sum: +0.51) and weakest in Amber m^6A (sum: +0.26), with the values for the unmodified base in between. It is thus questionable how much the heritage of the FF is actually visible in these extensions, and how much volatility is introduced by the mapping of QM properties to fixed, partial charges,[3] especially in delocalized ring systems. In other words, it is possibly more appropriate to refer to any FF that has been amended in this way as a “hybrid FF.”

Details of progress index-guided sampling (PIGS)

All details on how PIGS operates can be found in the original publication,[4] but, for added clarity, we repeat some salient points here. As an adaptive sampling technique, PIGS propagates multiple replicas under identical conditions: the ambient conditions (temperature, density), the system composition, the potential energy function, and all other simulations settings are identical across replicas. At a fixed interval, some copies might be terminated, and their coordinates might be replaced with those of another replica. This termination/duplication scheme is *per se* lossy and has no benefit over keeping the replicas independent of one another unless the decision to terminate a replica is done in a targeted manner. To make the algorithm both scalable and compatible with high-performance computing (HPC) settings, the number of total copies is fixed

and the cost of evaluating the reseeding criterion (see next paragraph) is kept constant. The data produced by a PIGS run can be viewed as an ensemble of short trajectories with a strict, tree-like topology when looking forward in time. Replicas can only be terminated (dead branch) or grow (spawning a new branch), but two branches can never coalesce or swap. This means that every point in time can be uniquely tracked back to a specific starting point (and replica), which implies that there is a geometrically continuous path in time from the very beginning for all simulation snapshots. This last point is an important consideration for the construction of transition state networks.

As mentioned in the main text, PIGS uses a particular feature space to define a (Euclidean) measure of distance within. Throughout any stretch between two reseeding intervals (here, 100 ps), all replicas (here, 64) record their feature data at a finer interval (here, 1 ps, accumulating 64000 data points per stretch). Since we rely on a GROMACS-CAMPARI hybrid approach for PIGS,[5] this entails intermittently writing compressed trajectories at 1 ps time resolution and analyzing them through individual invocations of CAMPARI: using the aforementioned measure of distance, the progress index algorithm[6] determines an ordering of all the 64000 snapshots in a way that makes self-similar snapshots appear near each other. These data are used to rank the replicas' snapshots populated at the end of the reseeding stretch in question by redundancy, from low to high. This ranking is composite and uses 3 separate criteria: the distance from any other terminal snapshot (larger is better), the position in the progress index (larger is better), and the (approximate) minimum geometric distance to any snapshot with smaller progress index value (larger is better). All 3 criteria are engineered to heuristically identify terminal snapshots that are maximally different from the 63 others, *i.e.*, to penalize redundancy. A fixed number of replicas (here, 32) with the best ranks are deemed "protected." They are used to stochastically reseed (*i.e.*, terminate and replace) replicas with lower ranks (more likely for higher rank differential). Because any reseeding is *per se* lossy, this strategy becomes problematic once there is no redundancy left. To avoid negative consequences from this, a replica is always protected, irrespective of rank, if its snapshots form a relatively compact group in the progress index. This is typically the state reached by a PIGS simulation toward the end (compare Fig. S6 below), where the reseeding rate has dropped and the uninterrupted trajectory segments can become very long, which is good for Markov state model (MSM) construction.

Transition path theory (TPT)

To appreciate the nature of the challenge we try to address using MSMs and TPT here, it is illustrative to start with a Kramers view.[7] Suppose that there are two main states with a large barrier separating them so that there is a separation of time scales for relaxation within a state

relative to crossing the barrier. This system is of interest because a similar two-state assumption has to be imposed onto many experimental results, such as binding assays. The crossing rate depends on height and other factors but is computable meaningfully only if the underlying system evolves stochastically, with diffusion-like motion (fluctuation-dissipation).[8] Complex dynamical systems at the molecular scale, like biopolymers, often appear to evolve stochastically, and these dynamics can often be approximated in a memoryless (overdamped) limit. In a space of discrete rather than continuous states, MSMs are the canonical way of modeling this limit.[9]

However, a typical MSM, which is defined by its transition matrix, has many more than two states. If we assume that we know which states to lump into the two boundary states, any MSM allows simulation-based predictions of both kinetics and thermodynamics: so-called random walkers[10] can be initialized in state A according to their steady-state population (equilibrium distribution), and one can simulate their progression through the transition state network until they reach state B. If the distribution of arrival times is exponential, a single-exponential rate is fittable. If it is not, the scenario is most likely not Kramers-like (multiple barriers, or no separation of timescales). The first complexity is to understand whether boundary states A and B are kinetically homogeneous. For example, as a thought experiment, imagine that m^6A and one of the aromatic side chains of the cage can undergo a concerted flip. If this flip is slow but both sub-states are part of the bound state, an error will be introduced since two processes are integrated into one rate. For the random walkers, this would mean that distinct distributions of arrival times are (incorrectly) treated as statistically homogeneous.

The random walkers will also sample a specific path. Outside of extremely simple MSMs, these paths will all be different and all individually have vanishing probability. The path probability is simply the joint probability, *i.e.*, the product of the transition matrix elements that were picked. The paths are stochastic: they contain detours, entering and returning from dead ends, flickering between two adjacent states, waiting in a state, *etc*. Still, one might expect that there are subfamilies of paths that share certain properties, such as characteristic sets of states that are visited. For example, if the m^6A binding site could be reached from two sides, one would expect that there are two major families of unbinding pathways, and the random walkers should reflect this. We attempted such an analysis, but clustering a very large number of random walker paths and plotting them on the network allowed no straightforward pathway delineation.

If a state-centric view is adopted instead, we bridge over into TPT.[11,12] Suppose that whenever a state is visited by a random walker, it is recorded whether this walker reached the target state before it returned to the source state or not. The fraction of walkers reaching the target first, relative to all walkers passing through the state, is known as the committor probability, and it can

be calculated without having to enumerate random walkers. It is, arguably, the most natural progress variable (or reaction coordinate)[13] for the two-state reaction we are trying to describe. Given states *B* (the target), *U* (the source), and *I* (everything else), the plus or forward committor, q_i^+, is the solution to:

$$-q_i^+ + \sum_{j\in I} T_{ij} q_j^+ = -\sum_{j\in B} T_{ij} \qquad \text{(S1)}$$

Where the T_{ij} are the transition matrix elements. The minus (or backward committor) is the analogous solution to:

$$-q_i^- + \sum_{j\in I} \left(\frac{\pi_j}{\pi_i}\right) T_{ji} q_j^- = -\sum_{j\in U} \left(\frac{\pi_j}{\pi_i}\right) T_{ji} \qquad \text{(S2)}$$

Both committor values allow ordering the states comprising *I* by their proximity to the boundary states. They are always related as $q_i^+ \approx 1 - q_i^-$, but the relation is exact only if the transition matrix fulfills detailed balance. It is intuitive that two connected states that differ in q_i^+ provide information on which direction the target state is in. This is the logic of a flux network (eqs. 1 and 2 in the main text), and it is illustrative to consider the detailed balance limit:

$$f_{ij} - f_{ji} = \pi_i T_{ij} \left(1 - q_i^+\right) q_j^+ - \pi_j T_{ji} \left(1 - q_j^+\right) q_i^+ = \pi_i T_{ij} \left(q_j^+ - q_i^+\right) \qquad \text{(S3)}$$

The second equality also follows because of detailed balance per its definition: $\pi_i T_{ij} = \pi_j T_{ji}$. The sign of the flux will mirror exactly the sign of the difference in committor values. Thus, the "productive" flux, f_{ij}^+, considers only transitions where the net flux is positive (eq. 2 in the main text).

The task of decomposing the total flux into pathways is equivalent to the task of finding high-capacity paths in the flux network, which can be easily recast and solved as a shortest-path problem, here using Eppstein's algorithm[14] as implemented in CAMPARIv5 (https://campari.sourceforge.net/V5). Even though these are no longer stochastic paths a random walker would take (they can only advance in terms of committor), the combinatorial growth of their number generally precludes even an extremely large number of paths from amounting to a substantial fraction of the total flux (eq. 3 in the main text). We tried this for our networks, but neither did the integrated flux reach reasonable values nor did the top-ranked paths show any particular grouping. The most common workaround to this problem stipulates that paths can be clustered implicitly by modifying the flux network continuously. Specifically, the total[11] (or a significant fraction[15] of the) capacity of the bottleneck transition along the pathway is removed, and the updated flux network is used in the next step, effectively eliminating all (or most) paths using this transition. This makes several assumptions, which we did not see fulfilled

here: that there is a clear, single bottleneck on a given path; that the bottleneck is unique to a family of paths, *etc*.

In summary, the structure of the problem as two-state and modelled by a memoryless stochastic evolution remains largely an imposition, yet both impositions are somewhat inevitable. The Markov assumption only ever holds approximately, but more general frameworks require even more data, limiting the scope of reachable conclusions. Furthermore, our TPT-based analysis as well as other approaches[16] have highlighted that the dynamical behavior of molecular systems often markedly deviates from models involving two clearly delineated boundary states, at least at the spatial and temporal resolutions we have access to here. It is, in particular, difficult to define a kinetically homogeneous disordered (here, unbound) state. Such homogeneity is an imposition and not generally an accurate reflection of the biomolecular systems under study. Nevertheless, by resorting to the two-state assumption, we can assure that compact results are produced, which are, in many cases, directly comparable to experiment. Finally, robust diagnostics of the quality of the model are a research field on their own: here, we employ the Kemény-constant, which quantifies the expected timescales of dynamics on a Markov chain,[17,18] and a straightforward variance/robustness estimation through subsampling of the count matrix.

Supplementary Tables

Systems shared	Clusters	Frames
**4**	1013	811779
**3**	433	312380
**2**	246	23871
**1**	262	3586
**Total**	1954	1151616

Table S1. **Number of clusters and snapshots (frames) shared by systems**.

System	Clusters	Total frames	Clusters ***B***	Clusters ***U***	Frames ***B***	Frames ***U***
**AAA**	1627	256K	13	253	20277	95971
**A*m*⁶AA**	1460	256K	8	275	43373	61310
**GAC**	1601	256K	13	279	43405	84138
**G*m*⁶AC**	1417	384K	9	245	66575	33532

Table S2. **Splits of cluster and snapshot (frame) numbers across systems.** Totals (columns 2 and 3) are presented along with the number of clusters and snapshots (frames) defining the boundary states *B* and *U* for every system.

Parameter	CHARMM36	Amber
vdw-modifier	force-switch	potential-shift
rlist	12 Å	9 Å
rvdw	12 Å	9 Å
rvdw-switch	10 Å	n/a
rcoulomb	12 Å	9 Å
dispcorr	no	no
thermostat coupling time	1 ps	1 ps
box shape	rhombic dodecahedron	truncated octahedron
minimum solute-to-box distance	1.2 nm	1.2 nm
water molecules	~2700 TIP3P	~2900 OPC

Table S3. **FF-specific Gromacs run parameter and setup choices used in simulations of the pentanucleotide in water**. Values that were shared across all MD simulations in the work can be found in Table S4.

Parameter	Value
ensemble	NVT
thermostat	Bussi-Parrinello
cutoff-scheme	Verlet
vdwtype	cut-off
coulombtype	PME
PME order	4
PME spacing	0.12 nm
ewald-rtol	10^{-5}
geometric constraints	bonds involving H
constraint algorithm	LINCS
lincs-order	4

Table S4. **Universal Gromacs run parameter choices used in simulations of both the pentanucleotide in water and the complexes**.

System	Pentanucleotide in water (all conditions)	Complex Am^6AA	Complex AAA	Complex GAC	Complex Gm^6AC
**Avg. *T* (K)**	300.0	300.3	301.7	302.1	302.1
**SD (K)**	0.0	0.6	1.3	1.2	1.2

Table S5. **Average temperatures and their standard deviation (SD) across replicas for different runs.** Because the complex simulations use PIGS, we retained energy data only for one reseeding cycle. The data in the table are based on a single stretch of only 100 ps (50 samples) near 50 ns simulation time, but for 64 replicas (6.4 ns cumulative). In contrast, the data for the pentanucleotides in water are based on the entire sampling of 16x200 ns, which is why the SD across the 16 replicas is tiny.

Residue
Asn363*
Asn364*
Leu380
Pro381
Val382
Asn383
Pro431*
Ala432*
Gly433*
Met434*
Ser435
Ala436
Lys437
Met438*
Leu439

Table S6: **Protein residues, for which ϕ- and ψ-angles serve to diversify the protein-RNA complex from its initial, bound configuration in PIGS.** Residues not marked with an asterisk are included only from 50 ns onwards. The numbering is the same as that in PDB entry 4R3I.

Supplementary Figures

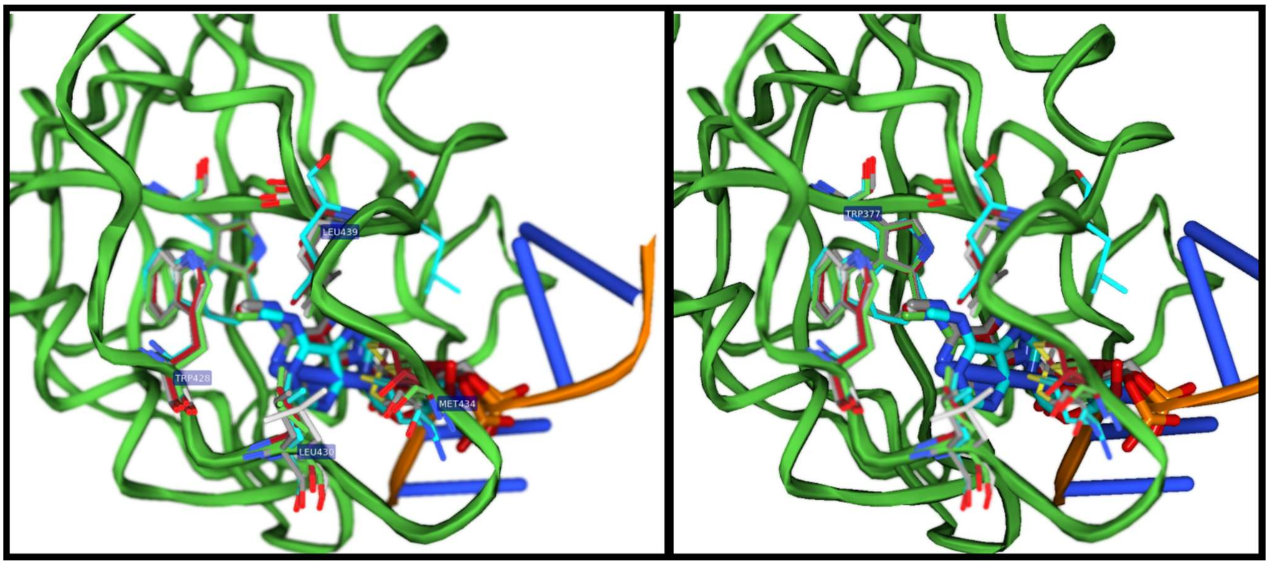

Figure S1. **Overlay of the binding pose of the modified base in several experimental *holo* structures**. The structures corresponding to 4R3I (green), 6ZCN (chain B; red), 7L4X (dark grey), 6RT6 (chains B,E; light grey), and 2MTV (model #1; cyan) were aligned and rendered (binding site residues as thin sticks, the modified base as thick sticks, and the backbone of 4R3I as Cartoon, RNA in orange/blue). The left side emphasizes the foreground (with Trp428 and the binding loop residues Leu430, Met434, and Leu439 labelled) while the right panel emphasizes the methylated base and Trp377 in the back. Except for Met434, even the side chains show little variability.

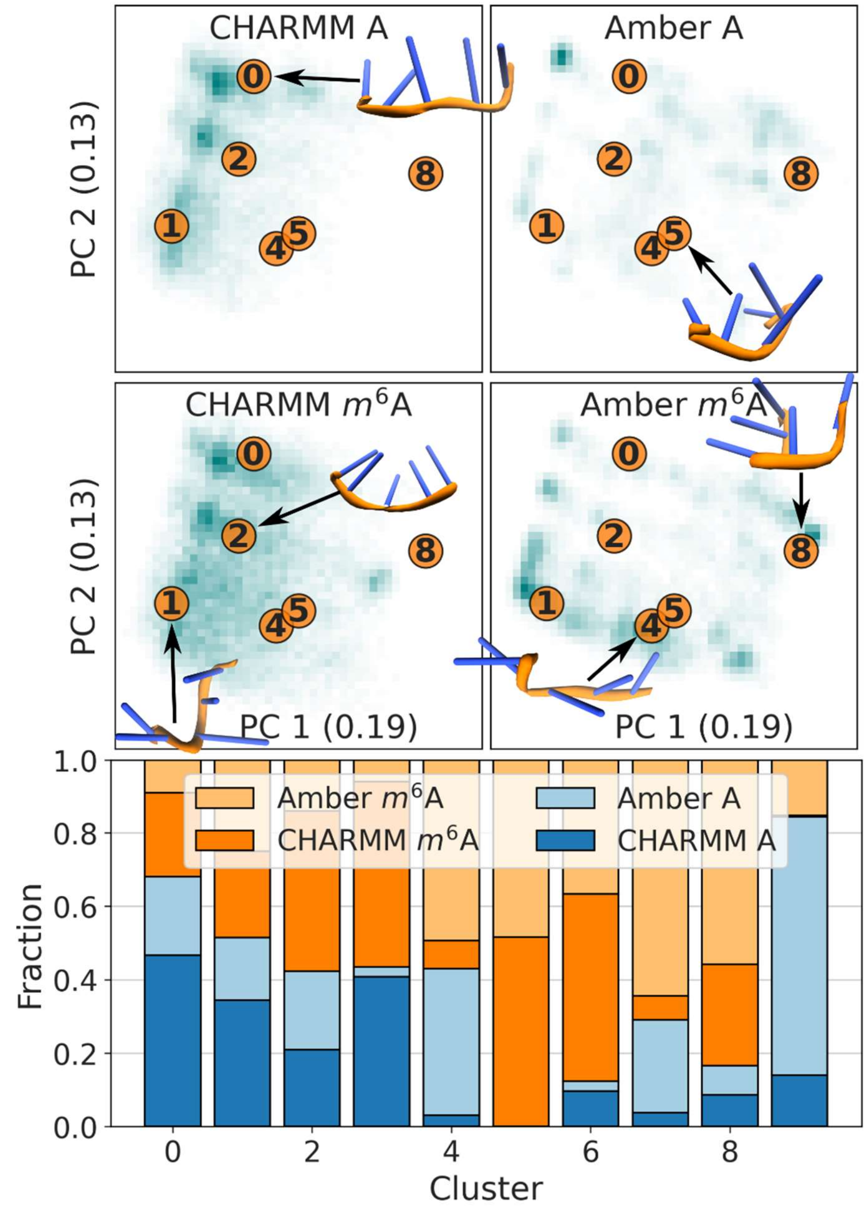

Figure S2. **Principle component analysis based on all 31 non-redundant dihedral angles of the pentanucleotide in water**. Data from both FFs and methylation states are included. **Left**. The fraction of variance preserved as a function of the number of retained components. **Right**. Color map of the PCA loadings for the first ten PCs. Orange lines and Greek letters mark the RNA angles the loadings act on.

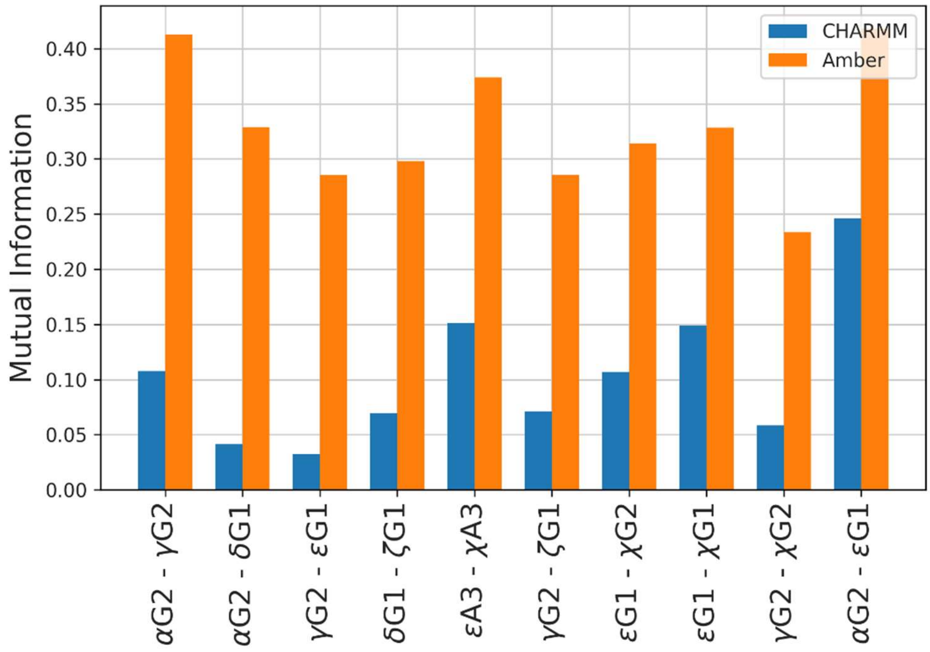

Figure S3. **Mutual information for the 10 non-adjacent dihedral angle pairs of the methylated pentanucleotide in water**. Data are shown for those pairs of dihedral angles where mutual information differs the most between force fields.

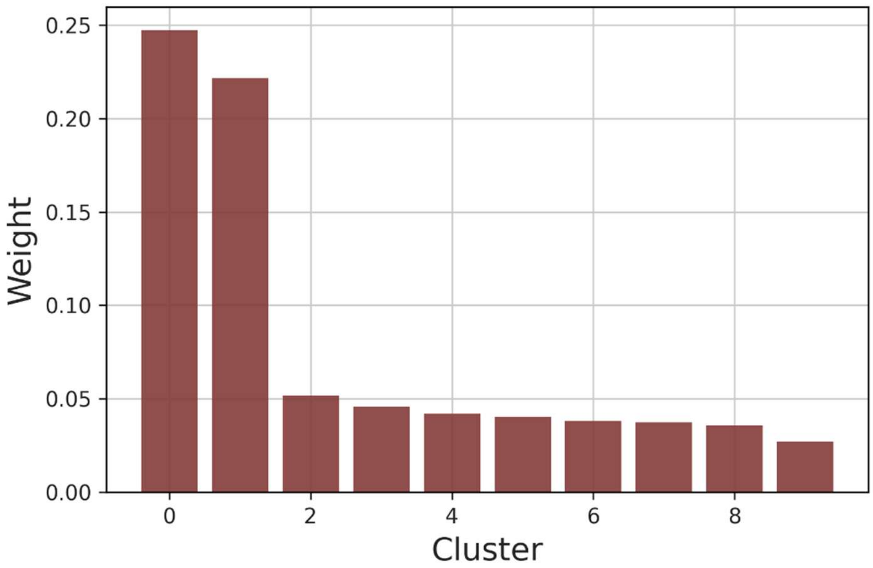

Figure S4. **Population of the 10 most populated clusters determined for the pentanucleotide in water**. The clusters are based on 31 dihedral angles projected onto 10 principal components. The partitioning is calculated for both force fields and methylation states jointly.

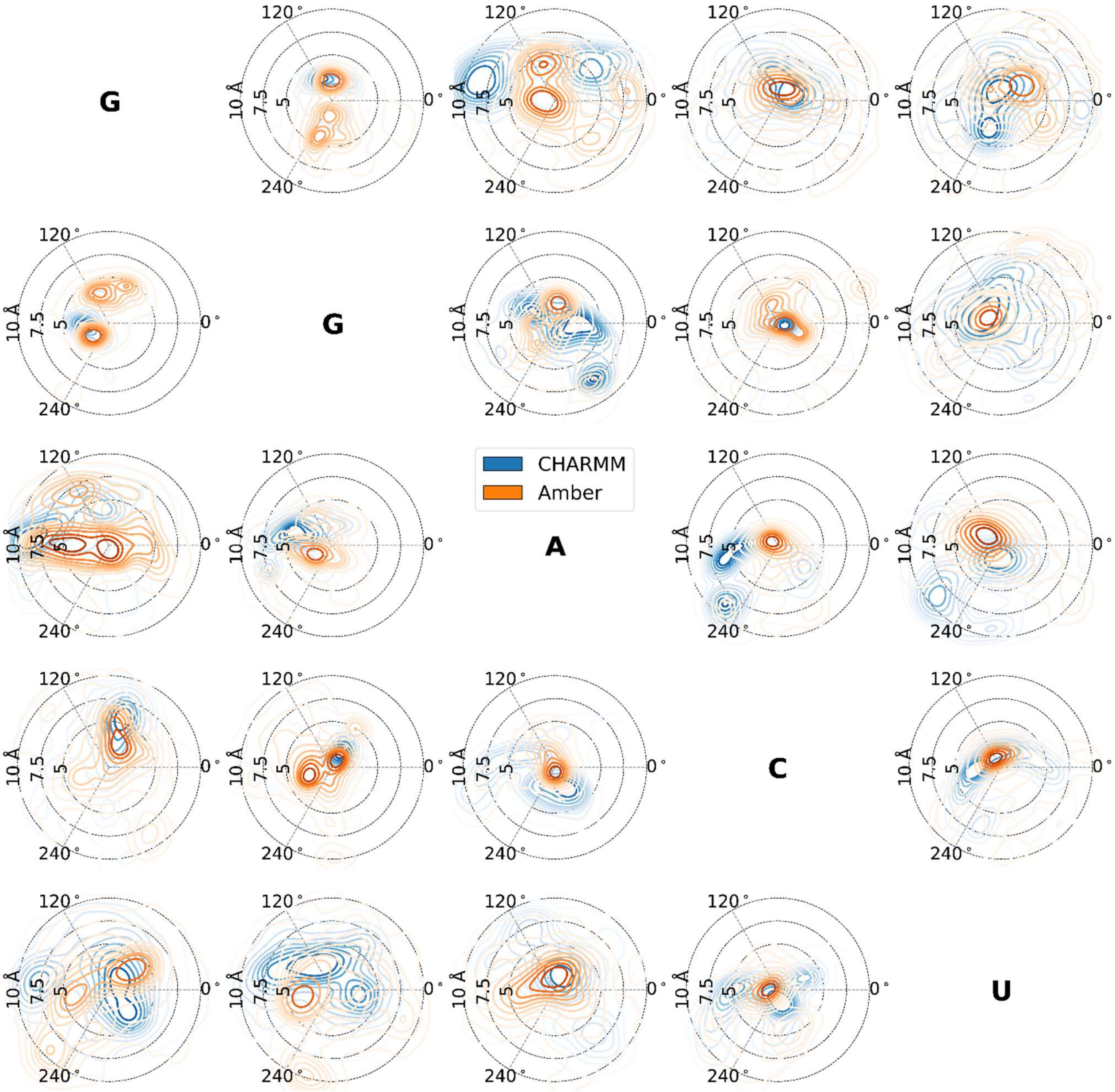

Figure S5. **Comparison of the base stacking in the methylated pentanucleotide in water for Amber and CHARMM force fields**. A kernel density estimate of the probability density is shown. Each subplot represents the stacking for one nucleotide pair.

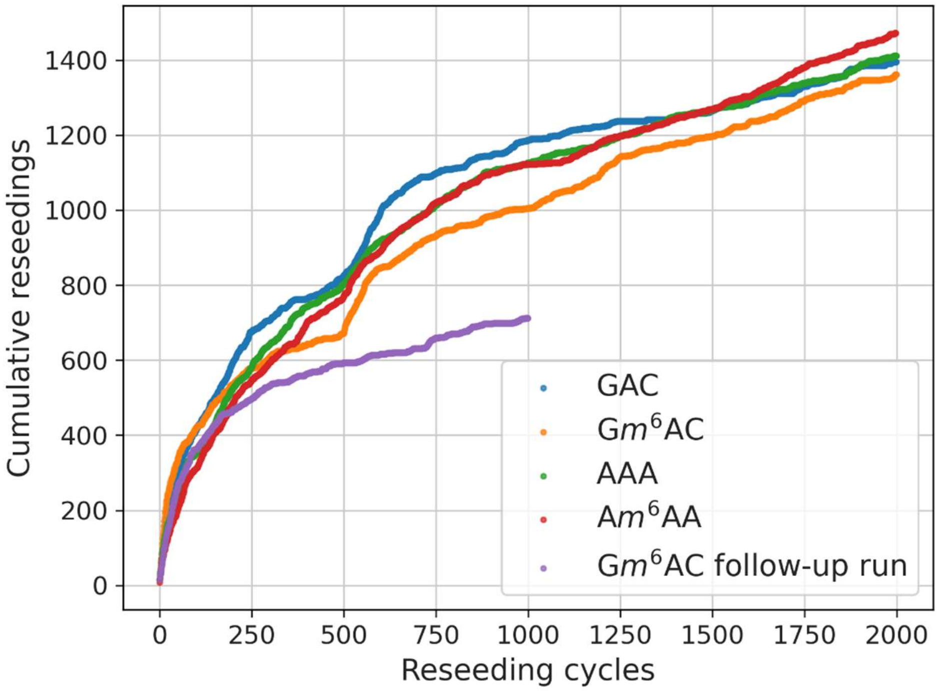

Figure S6. **Cumulative reseedings as a function of reseeding cycles of 100 ps for each of the five PIGS ensembles**. As diversification of the feature space proceeds, the rate drops because of the uniqueness override in the PIGS heuristic (see above and original publication).[4] Thus, a plateauing of this rate is a direct indicator of diversification.

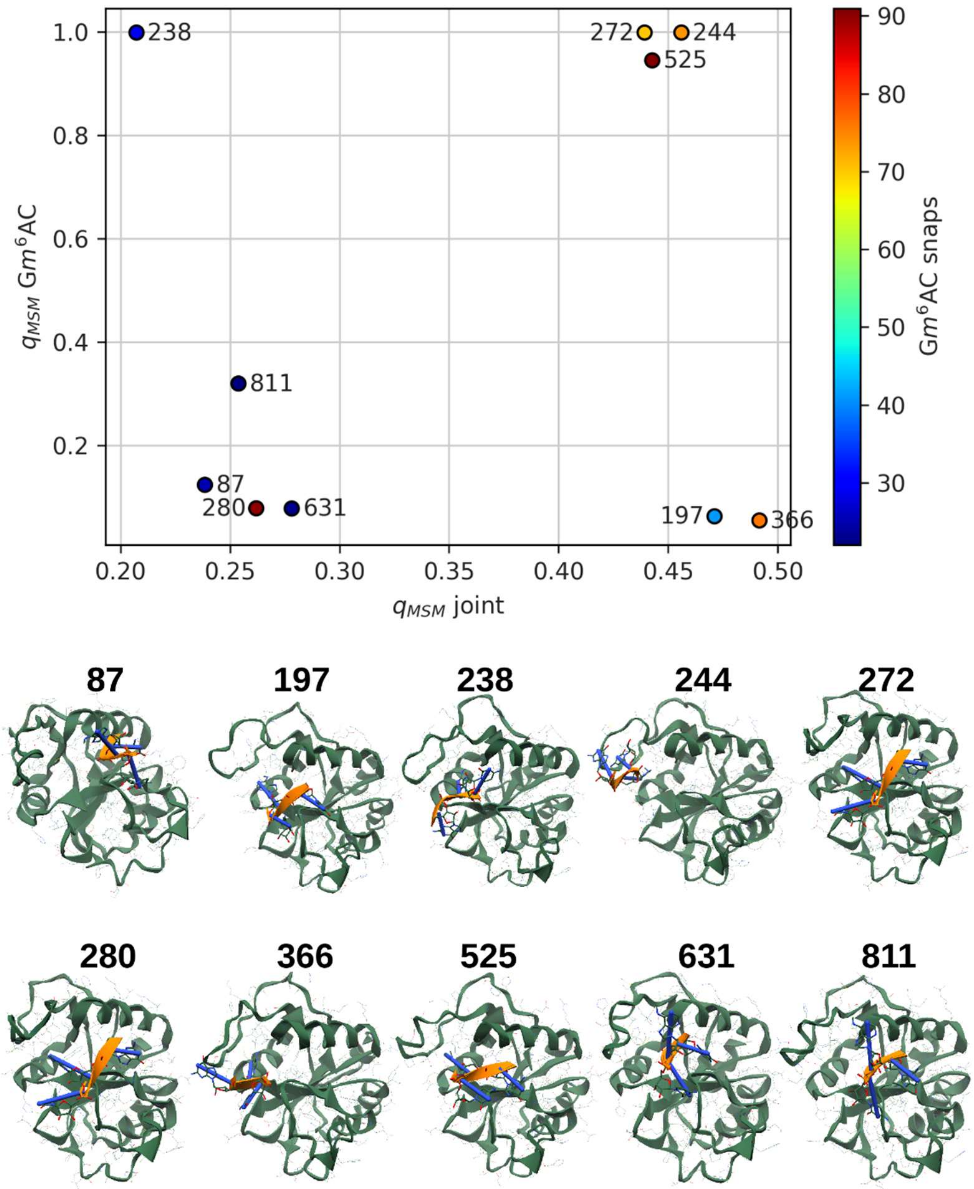

Figure S7. **Clusters selected for additional sampling of G*m*^{6}AC**. A preliminary MSM constructed for all four systems jointly resulted in committor values of less than 0.5 for these (see main text). Top: The committor calculated from a G*m*^{6}AC-specific model is shown on the y-axis. Based on this analysis, structures, which had been observed to be productive only in control simulations, were included to increase the diversity of the committor-based PIGS ensemble. Additionally, the selected clusters were required to contain at least 20 configurations from G*m*^{6}AC, which is encoded by the color of the circle. The cluster index is annotated (cf. Fig. S8). Bottom: Representative cartoon images for the clusters referenced above. The protein domain is in green with the RNA in orange/blue. The orientation and viewing angle are comparable for most renderings.

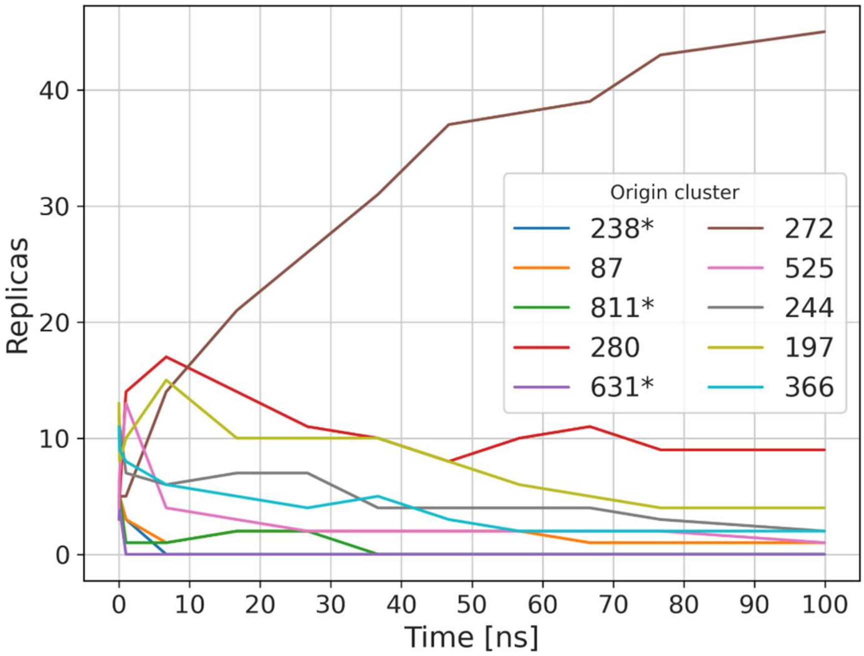

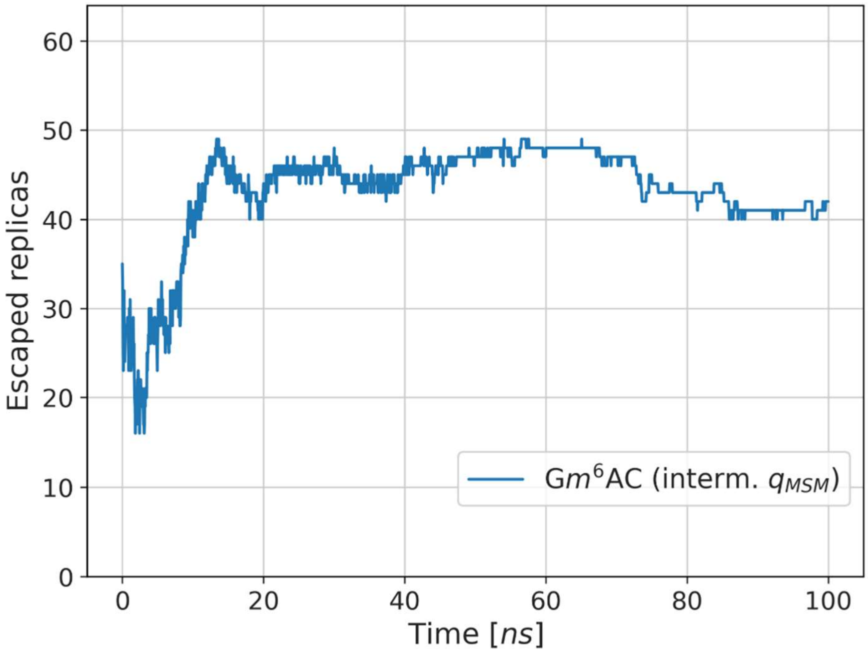

Figure S8. **PIGS ensemble initiated based on the committor of a preliminary MSM**. **Top**. Number of replicas originating from a specified cluster (cf. Fig. S7). Due to the adaptive termination and duplication of replicas, not all clusters can or should necessarily survive the full simulation time (those marked with an asterisk did not). **Bottom**. The time-resolved number of replicas that exceed the distance threshold for successful unbinding of the central adenine from the aromatic cage (equivalent to Fig. 5 in the main text).

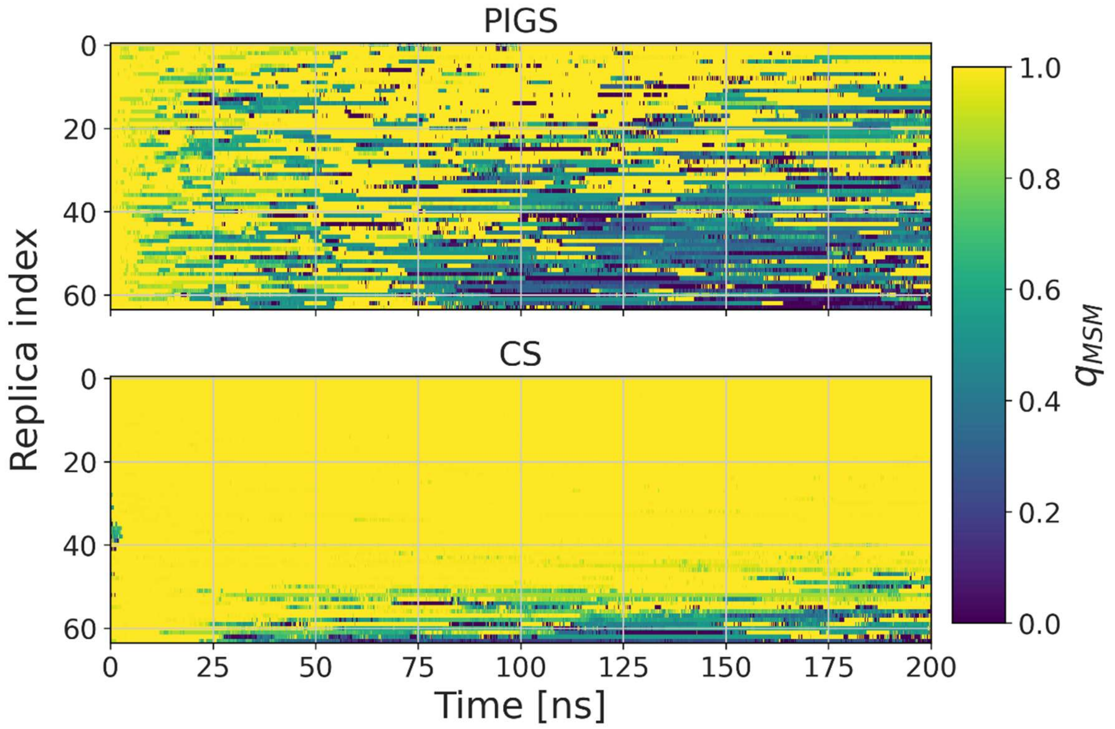

Figure S9. **Color map of committor values per replica**. The value of q_{MSM} is calculated from a joint MSM for CS and PIGS constructed specifically for the AAA system. The aggregate data are shown in Fig. 6 in the main text.

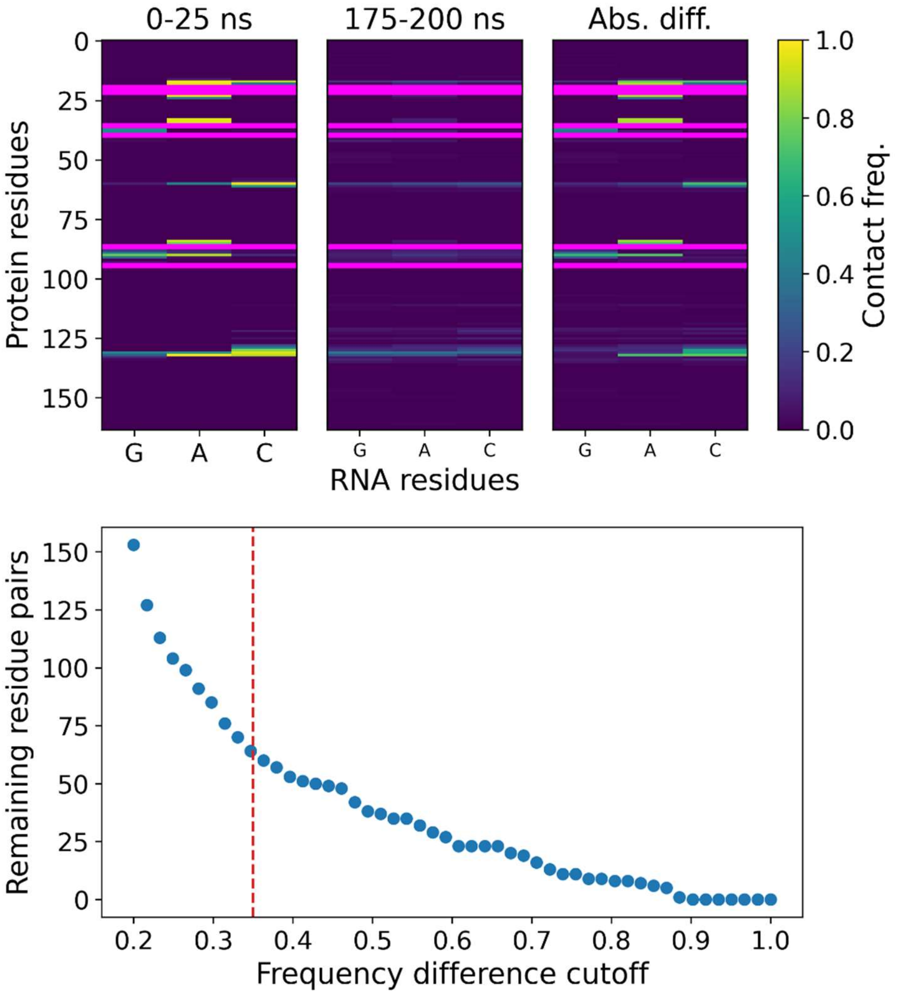

Figure S10. **Contact maps for GAC-YTHDC1**. **Top panels**. A contact is counted in a given molecular configuration if the smallest heavy atom distance between two residues is below 5 Å. Only intermolecular contacts are shown here, but all residue pairs are considered in the subsequent featurization. The protein residues selected for diversification in PIGS are highlighted by the magenta lines. Lighter squares indicate residue pairs considered for featurization. **Top left**. Contact map for the first 25 ns. **Top middle**. Contact map for 175-200 ns. **Top right**. Heat map of the absolute difference in contact frequencies. This was the map used to identify contacts carrying information on the unbinding process. **Bottom**. The number of remaining residue pairs as a function of the chosen cutoff in differential contact frequency based on contact maps of GAC at 0-25 ns and 175-200 ns.

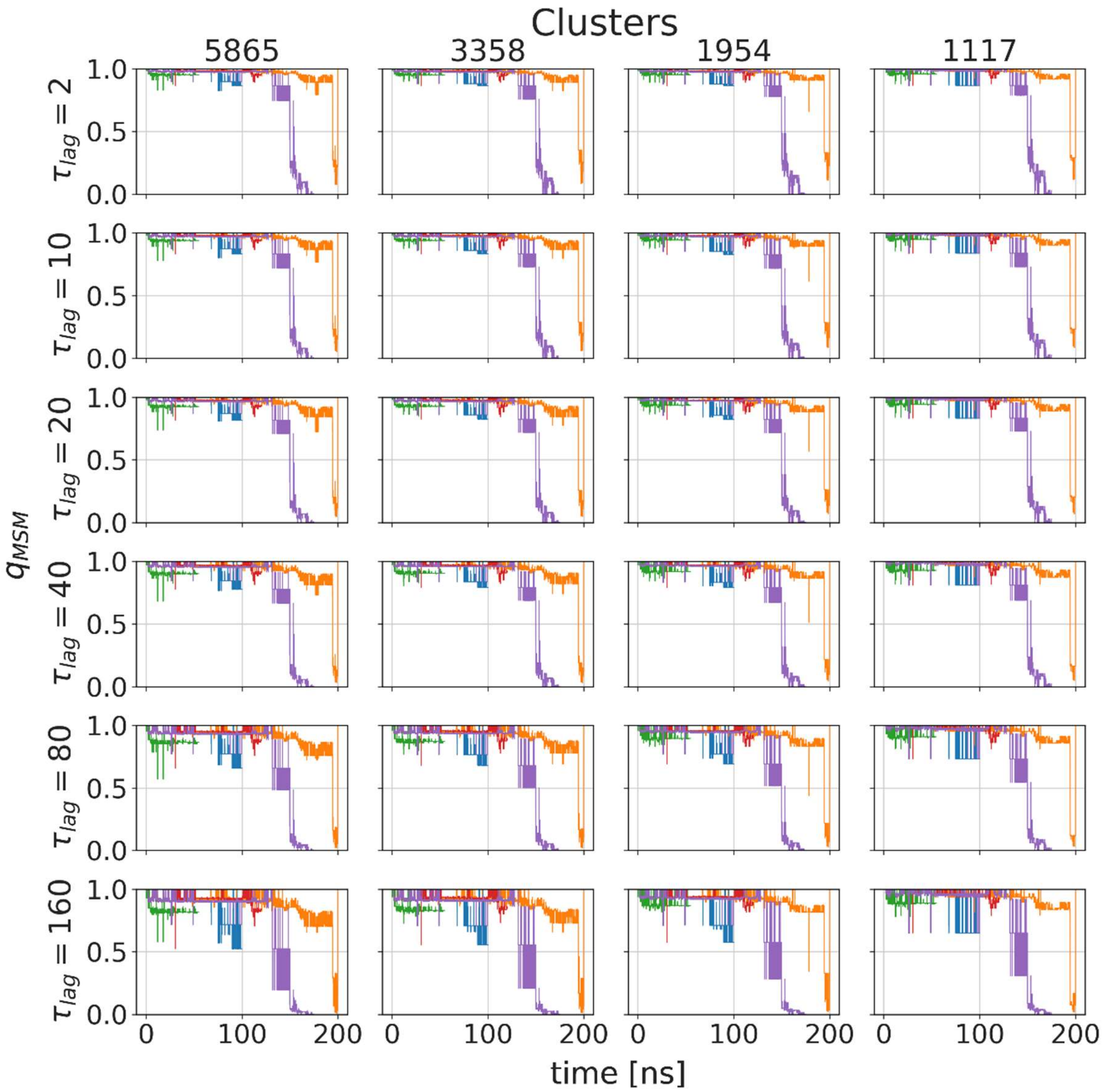

Figure S11. **Time evolution of committor values for several (partial) unbinding events**. The clustering resolution changes across columns (annotation on top) while the assumed lag time of the MSM (in multiples of 50 ps, same as Fig. S12) changes across rows (annotation on the left). Each panel contains several curves distinguished by color, each representing an individual unbinding or partial unbinding event tracked through the PIGS trajectory ensemble. The influence of both parameters on the committor values, q_{MSM}, is comparatively weak.

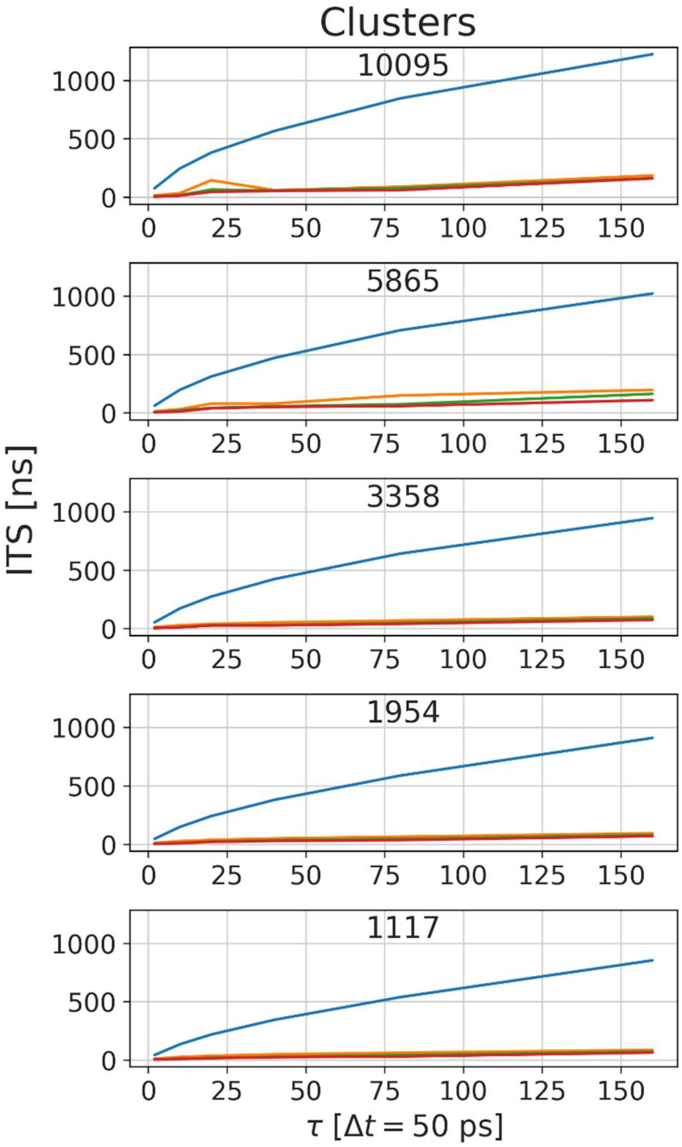

Figure S12. **Implied timescales for a MSM describing all four nucleotide systems jointly based on various clustering resolution**s. The four slowest timescales are shown in every panel and are distinguished by color. Data are plotted as a function of MSM lag time, covering the same range of values as Fig. S11. The different panels correspond to different clustering resolutions with the bottom four being the same as those shown in Fig. S11.

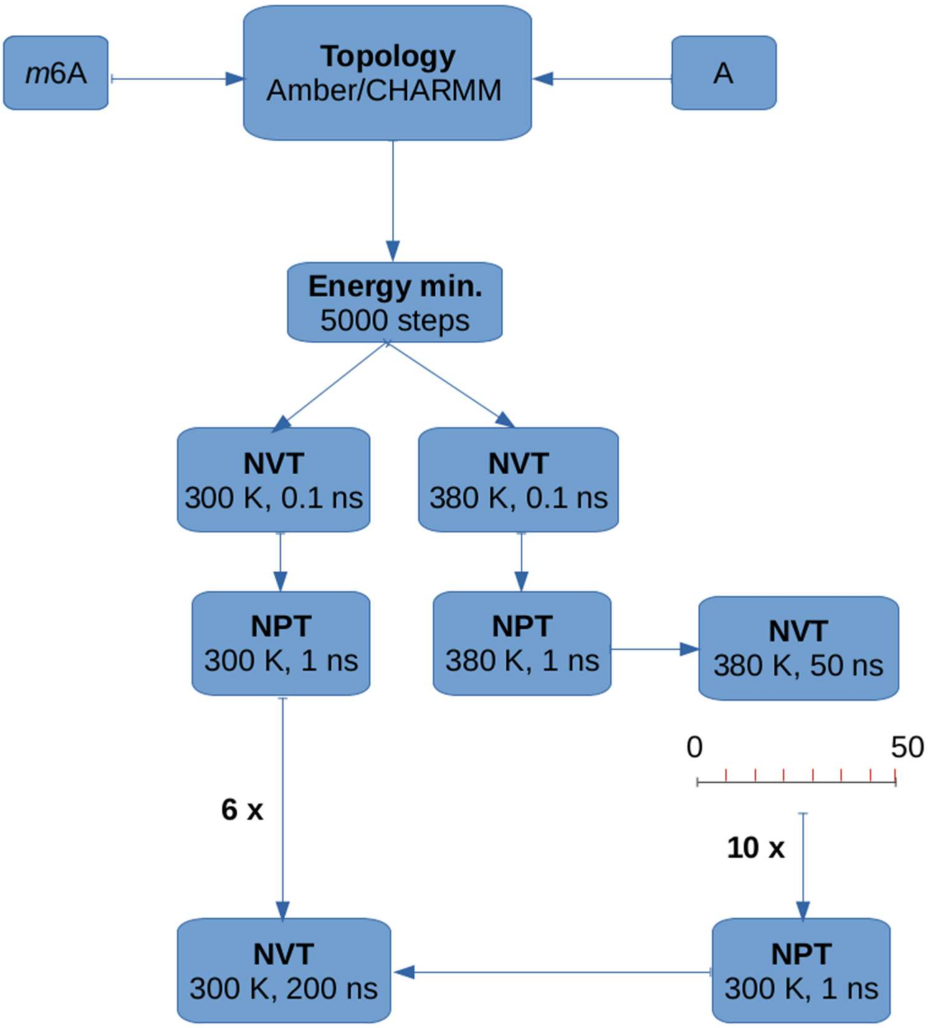

Figure S13. **Flow chart of system preparation for pentanucleotide runs**. GGm^6ACU and GGACU were each prepared both using Amber and CHARMM36 forcefields. Each of the four systems was simulated in 16 replicates of 200 ns each.

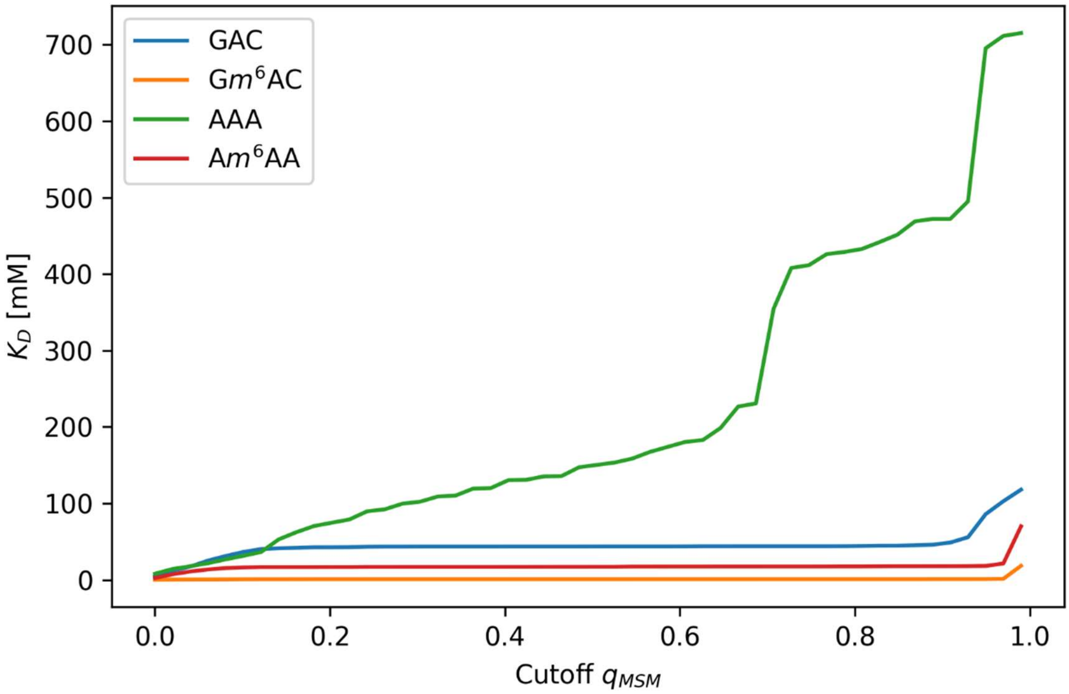

Figure S14. **Sensitivity of the equilibrium dissociation constant K_D**. Data are plotted as a function of the applied cutoff to determine the fraction of the intact complex, and subsequently the effective concentration of [*LR*], [*L*], and [*R*].

Supplementary References

1. Krepl, M., Damberger, F. F., von Schroetter, C., Theler, D., Pokorná, P., Allain, F. H. T., and Šponer, J. (2021) Recognition of N6-Methyladenosine by the YTHDC1 YTH Domain Studied by Molecular Dynamics and NMR Spectroscopy: The Role of Hydration. *J. Phys. Chem. B* **125**, 7691--7705
2. Xu, Y., Vanommeslaeghe, K., Aleksandrov, A., MacKerell, J. A. D., and Nilsson, L. (2016) Additive CHARMM force field for naturally occurring modified ribonucleotides. *J. Comput. Chem.* **37**, 896--912
3. D'Esposito, R. J., Myers, C. A., Chen, A. A., and Vangaveti, S. (2022) Challenges with Simulating Modified RNA: Insights into Role and Reciprocity of Experimental and Computational Approaches. *Genes* **13**, 540
4. Bacci, M., Vitalis, A., and Caflisch, A. (2015) A molecular simulation protocol to avoid sampling redundancy and discover new states. *Biochim. Biophys. Acta* **1850**, 889--902
5. Bacci, M., Langini, C., Vymětal, J., Caflisch, A., and Vitalis, A. (2017) Focused conformational sampling in proteins. *The Journal of Chemical Physics* **147**, 195102
6. Blöchliger, N., Vitalis, A., and Caflisch, A. (2013) A scalable algorithm to order and annotate continuous observations reveals the metastable states visited by dynamical systems. *Comput. Phys. Commun.* **184**, 2446-2453
7. Hänggi, P., Talkner, P., and Borkovec, M. (1990) Reaction-rate theory: fifty years after Kramers. *Reviews of Modern Physics* **62**, 251-341
8. Kubo, R. (1966) The fluctuation-dissipation theorem. *Reports on Progress in Physics* **29**, 255
9. Husic, B. E., and Pande, V. S. (2018) Markov State Models: From an Art to a Science. *Journal of the American Chemical Society* **140**, 2386-2396
10. Masuda, N., Porter, M. A., and Lambiotte, R. (2017) Random walks and diffusion on networks. *Physics Reports* **716-717**, 1-58
11. Berezhkovskii, A., Hummer, G., and Szabo, A. (2009) Reactive flux and folding pathways in network models of coarse-grained protein dynamics. *J. Chem. Phys.* **130**, 205102
12. Metzner, P., Schütte, C., and Vanden-Eijnden, E. (2009) Transition path theory for Markov jump processes. *Multiscale Modeling & Simulation* **7**, 1192-1219
13. Krivov, S. V. (2011) Numerical Construction of the pfold (Committor) Reaction Coordinate for a Markov Process. *The Journal of Physical Chemistry B* **115**, 11382-11388
14. Eppstein, D. (1998) Finding the k Shortest Paths. *SIAM J. Comput.* **28**, 652--673
15. Bacci, M., Vymětal, J., Mihajlovic, M., Caflisch, A., and Vitalis, A. (2017) Amyloid β Fibril Elongation by Monomers Involves Disorder at the Tip. *J. Chem. Theory Comput.* **13**, 5117--5130
16. Widmer, J., Langini, C., Vitalis, A., and Caflisch, A. (2023) Optimized reaction coordinates for analysis of enhanced sampling. *J. Chem. Phys.* **159**, 015101
17. Kemeny, J. G., and Snell, J. L. (1976) *Finite Markov Chains*, Springer-Verlag, New York
18. Koskin, V., Kells, A., Clayton, J., Hartmann, A. K., Annibale, A., and Rosta, E. (2023) Variational kinetic clustering of complex networks. *J. Chem. Phys.* **158**, 104112
